# Characterization and genomic analysis of the lytic bacteriophage vB_EclM_HK6 as a potential approach to biocontrol the spread of *Enterobacter cloacae* contaminating food

**DOI:** 10.1186/s12866-024-03541-9

**Published:** 2024-10-15

**Authors:** Hasnaa R. Temsaah, Ahmed F. Azmy, Amr E. Ahmed, Hend Ali Elshebrawy, Nahed Gomaa Kasem, Fatma A. El-Gohary, Cédric Lood, Rob Lavigne, Karim Abdelkader

**Affiliations:** 1 Biotechnology and Life Sciences Department, Faculty of Postgraduate Studies for Advanced Sciences (PSAS), Beni-Suef University, Beni-Suef, 62511 Egypt; 2 Department of Microbiology and Immunology, Faculty of Pharmacy, Beni-Suef University, Beni-Suef, 62511 Egypt; 3 Department of Food Hygiene, Safety, and Technology, Faculty of Veterinary Medicine, Mansoura University, Mansoura, 35516 Egypt; 4 Department of Hygiene and Zoonoses, Faculty of Veterinary Medicine, Mansoura University, Mansoura, 35516 Egypt; 5 Laboratory of Gene Technology, Department of Biosystems, KU Leuven, Kasteelpark Arenberg 21, Louvain, 3001 Belgium; 6 Department of Biology, University of Oxford, Oxford, OX1 3SZ UK

**Keywords:** *Enterobacter cloacae*, Raw chicken fillets, Bacteriophage, Antibiotic resistance, Sodium nitrite

## Abstract

**Background:**

Increased prevalence of *Enterobacter cloacae* within food products underscores food as an underexplored reservoir for antibiotic resistance, thus requiring particular intervention. Bacteriophages have been explored as a promising approach for controlling bacterial growth in different matrices. Moreover, their specific interaction and self-replication, put them apart from traditional methods for controlling bacteria in different matrices.

**Methods:**

Sixteen *Enterobacter cloacae* strains were recovered from raw chicken. These strains were used to isolate bacteriophages using enrichment protocol. The broad-spectrum bacteriophage was evaluated in terms of thermal, pH, shearing stress and storge. Moreover, its infection kinetics, in vitro antibacterial activity, cytotoxicity were also assessed. Genomic sequencing was performed to exclude any potential virulence or resistance genes. Finally, the capability of the isolated phages to control bacterial growth in different chicken samples was assessed alone and in combination with sodium nitrite.

**Results:**

The lytic bacteriophage vB_EclM_HK6 was isolated and showed the broadest spectrum being able to infect 8/16 *E. cloacae* strains with a lytic activity against its host strain, *E. cloacae* EC21, as low as MOI of 10^–6^. The phage displays a latent period of 10 min and burst size of 115 ± 44 and resistance frequency of 5.7 × 10^–4^ ± 3.0 × 10^–4^. Stability assessment revealed a thermal tolerance up to 60 ˚C, wide range pH stability (3–10) and the ability to withstand shearing stress up to 250 rpm. HK6 shows no cytotoxicity against oral epithelial cells up to 10^12^ PFU/ml. Genomic analysis revealed a Strabovirus with total size of 177,845 bp that is free from known resistance and virulence genes. Finally, HK6 pretreatment of raw chicken, chicken nuggets and ready-made cheese salad shows a reduced bacterial count up to 4.6, 2.96 and 2.81 log-units, respectively. Moreover, combing HK6 with sodium nitrite further improved the antibacterial activity in both raw chicken and chicken nuggets without significant enhancement in case of cheese salad.

**Conclusion:**

Enterobacter bacteriophage vB_EclM_HK6 presents a safe and effective approach for controlling *E. cloacae* contaminating stored chicken food samples. Moreover, they could be combined with a reduced concentrations of sodium nitrite to improve the killing capacity.

**Supplementary Information:**

The online version contains supplementary material available at 10.1186/s12866-024-03541-9.

## Background

The unprecedented dissemination of multidrug-resistant pathogens underscores inadequate infection control practices that could necessitate more restrictive control measures. Normally, nosocomial infections are transmitted by contact with colonized healthcare providers or contaminated surfaces including medical devices and personal objects (bed sheets, pillows) [1]. Nevertheless, consuming water or food products contaminated with high bacterial load (e.g., raw, undercooked, without suitable preservatives) may be an additional route of infection, especially for hospitalized patients with weakened immune systems. This is further confirmed by high prevalence of nosocomial pathogens (non-foodborne pathogens) within different food products from animal or plant origins [2]. *Enterobacter cloacae* is a notorious opportunistic pathogen responsible for different nosocomial and community-acquired infections including bacteraemia, pneumonia, urinary tract infections, and intra-abdominal infections [3]. Management of *E. cloacae* infections is cumbersome, because of its resistance to different classes of antibiotics [4]. Besides, it is considered as reservoir for mobile genetic elements (plasmids, insertion sequences and integrons), thereby posing a hazard of sharing resistance genes with other relevant hospital pathogens [5, 6]. As ubiquitous organism [3], contamination with *E. cloacae* during food production chain is possible whenever rigorous sanitation measures are not followed. This concern is further supported by a growing body of reports describing recovery of *E. cloacae* from various animal-derived food products including cheese, yogurt, beef, chicken meat and neonatal feeding formula [5, 7, 8]. Serving contaminated food for immunocompromised patients may put them at risk of *E. cloacae* infection. Several studies have proposed that increasing intestinal bacterial load of *E. cloacae* may trigger subsequent systemic infection [9, 10]. Therefore, effective food biocontrol strategies should be explored to ensure keeping the bacterial count within safe levels.

Sodium nitrite (Sn) is a food additive commonly implemented in meat-related industries to enhance sensorial characteristics and to control bacterial growth. Nevertheless, its reported side effects may restrict their application within bacterial inhibitory concentration [11]. Excessive consumption of Sn is associated with potential heart diseases and colorectal cancer due to formation of nitrosamine upon reaction with certain food components [12]. On the other side, bacteriophages have been widely accepted as a food bio-preservative with reported efficacy against challenging food pathogens, *Salmonella spp*. [13], *E. coli* [14, 15] and *Listeria monocytogenes* [16]*.* Bacteriophages have unique features, setting them apart from conventional preservatives. For example, self-replicating ability of bacteriophages ensures their presence in sufficient concentration to sustain their efficacy against specific bacteria, offering a continuous and adaptive approach to bacterial control. Moreover, their specific interaction with their respective host minimizes any potential undesirable effect to either consumer or food product [17].

Bacteriophages targeting *E. cloacae* have been previously reported and evaluated both in vivo and in vitro without shedding light on their capacity to control bacterial growth in food products [18–20]. The main purpose of the current study is assessing the preservative capacity of the isolated phage (designed as HK6) against the food-recovered *E. cloacae* strains in different food matrices alone and in combination with sodium nitrite.

## Methods

### Bacterial isolation from food samples, identification and growth conditions

Thirty fresh chicken breast fillet samples were purchased individually over a period of 30 days from local supermarkets, located in Beni-Suef city, Egypt, and stored in ice. Samples were cut aseptically into equal pieces (10 g for each), transferred into flasks with 100 ml selenite broth and incubated at 37 ºC for 24 – 48 h with shaking (150 rpm). Samples showing bacterial growth (turbidity) were subsequently streaked over bile esculin agar plates (Oxoid, Basingstoke, Hampshire, UK) and incubated at 37 ºC for 24 h then inspected for black colonies. The positive colonies (black) were then identified up to species level using Microscan (Siemens Healthineers). All strains were sub-cultured from their respective bile esculin agar plates using LB (HiMedia Laboratories Private Limited, Maharashtra, India), incubated at 37°C with shaking (200 rpm), or streaked on LB agar plates (LB broth supplemented with 2% agar). Antibiotic sensitivity of the recovered *E. cloacae* strains was assessed against panel of eight antibiotics (Table 1) using conventional disk diffusion test with final bacterial inoculum density of 10^6^ CFU/ml.

**Table 1:** Overview of E. cloacae strains used in the current study. The table illustrates food origin, antimicrobial resistance and sensitivity to different bacteriophages (HK1 to HK6)

Phage sensitivity^b^								
*E. cloacae* strains	Food source	Antibiotic resistance^a^	HK1 sensitivity	HK2 sensitivity	HK3 sensitivity	HK4 sensitivity	HK5 sensitivity	HK6 sensitivity
EC3	Raw chicken fillet	AMP, AMC, CAZ, CXT	** + **	** + **		** + **	** + **	** + **
EC4	Raw chicken fillet	AMP, AMC. CXT						
EC5	Raw chicken fillet	AMP, AMC. CXT						
EC7	Raw chicken fillet	AMP, AMC, CAZ, CXT	** + **	** + **		** + **		** + **
EC10	Raw chicken fillet	AMP, AMC. CXT						
EC11	Raw chicken fillet	AMP, AMC, CAZ, CXT	** + **	** + **		** + **		** + **
EC12	Raw chicken fillet	AMP, AMC. CXT						
EC13	Raw chicken fillet	AMP, AMC. CXT						
EC16	Raw chicken fillet	AMP, AMC. CXT						
EC 21	Raw chicken fillet	AMP, AMC. CXT				** + **		** + EC**
EC 25	Raw chicken fillet	AMP, AMC, CAZ, CXT, CIP, SXT	** + **			** + **		** + **
EC 26	Raw chicken fillet	AMP, AMC. CXT						
EC 29	Raw chicken fillet	AMP, AMC, CAZ, CXT, CIP	** + **	** + **				** + **
EC 34	Raw chicken fillet	AMP, AMC, CAZ, CXT	** + **			** + **		** + **
EC35	Raw chicken fillet	AMP, AMC, CAZ, CXT		** + **				
EC38	Raw chicken fillet	AMP, AMC. CXT, CAZ, SXT	** + **	** + **	** + **	** + **		** + **

^a^*AMP* Ampicillin, *AMC* Amoxycillin – clavulanic, *CAZ* Ceftazidime, *CXT* Ceftriaxone, *CIP* Ciprofloxacin, *SXT* Sulfamethoxazole-trimethoprim

^b^** + **, phage sensitive (lysis zone)

### Bacteriophage isolation, purification and high titre preparation

Bacteriophages against *E. cloacae* were isolated using enrichment technique as described previously with minor modification [21]. Briefly, sewage samples were collected from different hospital and domestic sewage outlets, then centrifuged (6000 × g for 10 min) and the supernatants were then filtered using 0.45-μm polyvinylidene difluoride membrane filters (VWR, Leuven, Belgium). Afterwards, 40 ml of the filtered samples were mixed with 10 ml of 5 × LB and enriched with 100 µl of *E. cloacae* strains (up to eight strains) individually grown to exponential phase (OD_600 nm_ of 0.6) and incubated for 18–20 h at 37 ºC with shaking (100 rpm). Thereafter, the mixtures were centrifuged (6,000 × g for 10 min), filtered (0.45 µm) and collected individually. The collected supernatants were screened individually for potential bacteriophage lytic activity using spot on-lawn [22] and double layer [23] assays. Purification of individual bacteriophages was performed by repeated plating of each individual plaque up to seven times. Bacteriophage high titre stocks were prepared by collecting three lysis zones resulting from spot assay into 2 ml SM buffer (10 mM Tris–HCl, 10 mM MgSO4, and 100 mM NaCl [pH 7.5]). Bacteriophage titre was then determined using the double layer assay [23] and expressed in PFU/ml.

### Host range determination and transmission electron microscope imaging

The host spectrum of each individual bacteriophage was screened against a panel of sixteen *E. cloacae* strains (Table 1) using standard spot on-lawn assay. Concisely, 10 µl of a test bacteriophage (with final concentration of 10^5^ PFU/ml) were spotted over a surface of double layer agar individually lawn with *E. cloacae* strains (Table 1). The plates then incubated for 16—18 h at 37 ºC and observed for formation of lytic (clear) zones.

Morphological features of the isolated bacteriophage were examined using transmission electron microscope as described previously [24]. Ten microliters of the purified phage (with final concentration of 10^10^ PFU/ml) were placed over carbon-coated formvar/copper grids (200 mesh) and negatively stained with 2% aqueous uranyl acetate (pH 4.0) for 10 s. Then, observed with energy-filtering TEM (LIBRA 120, Carl Zeiss, Germany) at 120-kV accelerating voltage.

### Thermal, pH, shearing and storage stability assessment

The thermal stability of the isolated phage was assessed over a temperature range of 4 to 70 ºC as described previously [22]. Briefly, 1 ml of the phage stock (with final concentration of 10^10^ PFU/ml in SM buffer) was preincubated at each individual tested temperature for 1 h. subsequently, the residual phage titre was counted using double layer agar assay and *E. cloacae* EC21 as host strain. The assay was conducted as three independent replicates.

Similarly, the pH stability assay was conducted by diluting a phage stock (with final concentration of 10^12^ PFU/ml in SM buffer) in the Britton-Robinson universal buffer (0.04 M H_3_PO_4_, 0.04 M H_3_BO_3_, 0.04 M CH_3_COOH, and 0.15 M NaCl) adjusted to different pH values (3 to 10) to obtain a final phage stock of 10^10^ PFU/ml at each tested pH. Then, the prepared stocks were incubated at room temperature (25 ºC) for 1 h and the residual phage titre was determined using double layer agar assay and *E. cloacae* EC21 strain. Parallel controls were conducted by dropping 10 µl of the universal buffer with the respective pH.

The stability of the isolated phage towards shearing stress was evaluated by exposing phage particles (with final volume of 10 ml and concentration of 10^10^ PFU/ml) to shaking at four different rates (50, 100, 150, 200 rpm) for 30 min at room temperature. Then, the phage titre was determined using double layer agar assay and *E. cloacae* EC21 strain.

The storage stability of the phage suspended in SM was assessed over a period of six months as described before. The phage stock (with final titre of 10^10^ PFU/ml in SM buffer) was kept in fridge and its titre was determined weekly using double layer agar technique.

### One-step growth curve

Infection kinetics of the isolated phage was evaluated using a protocol for constructing one-step growth curve [22] and *E. cloacae* EC21 strain as a host strain. Briefly, an overnight culture of *E. cloacae* EC21 was grown in LB broth till reaching exponential growth phase (OD_600nm_ of 0.6), diluted to 10^8^ CFU/ml, then mixed with the purified phage (with final concentration of 10^6^ PFU/ml prepared in LB broth) to have a final multiplicity of infection (MOI) of 0.01. The mixture was then incubated at 37 ºC for 5 min for adsorption, then centrifuged (16,000 × g for 5 min) to collect the adsorbed phage. Subsequently, the pellets were re suspended in 10 ml LB broth and incubated at 37 ºC. Samples were taken at 5 min intervals for the first 35 min, followed by 10 min intervals till 90 min to quantify viable phage particles using double layer assay [23].

### In vitro antibacterial activity

The potential antibacterial activity of the isolated phage was evaluated in vitro using conventional spectrophotometry-based assay. An overnight culture of *E. cloacae* EC21 was 1:100 fold diluted in LB, then incubated at 37 ºC till reaching exponential phase (OD_600nm_ = 0.6). The obtained culture was then diluted to obtain 10^8^ CFU/ml, dispensed in 96-well plate and mixed with the tested phage to cover MOIs from 10^3^ to 10^–6^. Finally, OD_600nm_ was monitored at 60 min intervals over a period of 24 h using microtiter plate reader (Infinite 200 PRO Nano Quant; Tecan, Switzerland).

### Frequency occurrence of phage resistant mutants

Frequency of phage resistant-mutants development was investigated as described previously [25] using *E. cloacae* EC21 strain. Briefly, overnight culture of *E. cloacae* EC21 in LB was 1:100 fold diluted in LB broth and incubated at 37 °C with shaking (150 rpm) till reaching a final optical density of 0.6. Then, the culture was diluted to obtain a final density of 10^8^ CFU/ml and mixed with the purified bacteriophage (10^9^ PFU/ml in SM buffer) at MOI of 10, and incubated at 37 °C for 10 min. The mixtures were then added to 5 ml LB soft agar and plated over LB agar. The plates were then incubated at 37 °C for 16–18 h and examined by counting colonies. Controls were conducted by mixing bacterial culture with plain SM buffer. Frequency of phage resistant-mutants emergence are calculated dividing number of viable bacterial colonies appeared in the phage-treated culture by those appeared in SM treated one (control). Resistance of the survived bacterial colonies to HK6 was further confirmed using spot assay for a randomly selected 10 survived colonies.

### Bacteriophage genomic extraction, sequencing and in silico analysis

A high titre bacteriophage stock (10^12^ PFU/ml in SM buffer) was used for genomic extraction using the conventional phenol–chloroform/isoamyl alcohol method as described previously [26]. The concentration and purity of the recovered genome was assessed spectrophotometrically using nanodrop (Thermofischer; Waltham, MA, USA). DNA libraries were prepared with the Nextera Flex kit (Illumina, USA) and sequenced using the Illumina MiniSeq using a paired-end approach (2 × 150 bp). The reads were then controlled for quality using FastQC v0.12. and we used Trimmomatic v.0.39 [27] to remove adapter sequences, filter by length (> 50 bp), and trim lower quality regions. Finally, de novo genome assembly was performed using the SPAdes assembler v3.15.5 with default settings [28]. Assembly quality was inspected with Bandage [29] extract phage-related contigs.

The assembled bacteriophage genome was initially annotated using RAST online server [30]. The putative function of each annotated coding sequence was further confirmed using BLASTn online tool [31]. NCBI Conserved Domain Database (CDD) [32], HHpred [33] and InterPro Scan [34] servers were utilized to detect any conserved domains within the protein sequences encoded by each gene. Screening for t-RNA was performed using tRNAscan-SEv.2.0 [35]. Potential antibiotic resistance genes and virulence factors sequences were checked using Comprehensive Antibiotic Resistance Database (CARD) [36] and Resfinder [37] respectively. The lifestyle of the phage was predicted using artificial intelligence-based tools, PhageAI [38]. The phage genome was visualized using Proksee web-based tool (CG view) [39].

BLASTn analysis was performed to find bacteriophages showing high level of sequence similarity (> 95%). Bacteriophages with the highest nucleotide sequence similarities and query coverage (Top 14 hits) were retrieved form NCBI and used to generate intergenomic similarities heatmap using VIRIDIC (Virus Intergenomic Distance Calculator) online tool for taxonomical delineation [40]. Finally, the taxonomical delineation of the phage family was investigated by constructing proteomic tree using ViPTree [41]. The nucleotide sequence of the full bacteriophage genome was deposited in the NCBI GenBank database under accession number PP337149.

### Cytotoxicity assay against oral epithelial cells

Cell viability was assessed by the mitochondrial dependent reduction of yellow MTT (3-(4,5-dimethylthiazol-2-yl)-2,5-diphenyl tetrazolium bromide) to purple formazan as described previously [42]. Briefly, oral epithelial cells (purchased from National Research Centre, Cairo, Egypt) were maintained using DMEM-F12 medium, supplemented with 1% antibiotic–antimycotic mixture (10,000U/ml Potassium Penicillin, 10,000µg/ml Streptomycin Sulphate and 25µg/ml Amphotericin B) and 1% L-glutamine at 37 ºC under 5% CO_2_ for 10 days. Subsequently, cells with final density of 10^4^/well were dispensed in microtiter plate wells containing fresh media for additional 24 h under similar conditions. Then, the media was replaced with a fresh one, mixed with phage (or SM buffer in control) to reach a concentration range of 10^8^—10^12^ PFU/ml and left for 48 h. The media then was removed and 40 µl MTT (2.5 µg/ml) was added and incubated for 4 h at 37 °C. afterwards, the reaction was finalized by adding 10% sodium dodecyl sulphate followed by overnight incubation at 37 °C. Changes in cell viability, compared to SM treated cells, were observed colorimetrically at 620 nm using microplate multi-well reader (Bio-Rad Laboratories Inc., model 3350, Hercules, California, USA). IC50 and IC90 were calculated as the minimum phage concentration causing 50% and 90% cell viability reduction respectively.

### Bacterial count reduction assays in different food products

#### Food sample preparation

Solid (raw chicken fillet, processed chicken nuggets) and semi-solid (ready-made cheese salad) food samples were collected from local retail markets. To assure sterilization, the samples were washed with sterile distilled water for 5 min for decontaminating surface microorganisms then subjected to UV for at least 30 min [43]. Samples were then left for additional 5 min for drying and processed based on their texture. Solid samples were minced aseptically, while cheese salad samples were homogenized with sterile distilled water and placed in Petri dishes (five grams) in a biosafety cabinet. Finally, the samples were kept in fridge till use.

#### MIC analysis of sodium nitrite

The minimum inhibitory concentration (MIC) of Sodium nitrite was determined against *E. cloaca* EC21 strain using standard microdilution broth assay in Muller-Hinton (MH) broth. Briefly, *E. cloaca* EC21 was grown to exponential phase (OD_600nm_ of 0.6) in MH broth, diluted to 10^6^ CFU/ml and dispensed in 96-well microtiter plate. Subsequently, Sn (prepared as 10 mg/ml in MH broth) was mixed with bacterial culture to cover concentration range of 0.1—5 mg/ml. The plates then incubated at 37 °C for 16 -18 h and visualized for growth. Minimum inhibitory concentration (MIC) is the lowest concentration of Sn showing no growth.

#### Time-kill assay of bacteriophage in combination with sodium nitrite in food samples

*E. cloacae* EC21 strain was used to assess the antibacterial activity of bacteriophages individually and in combination of Sn in different food samples (raw chicken fillets, processed nuggets and cheese salad). The experiment was conducted by adding 1 ml of the purified bacteriophage (with final concentration of 10^12^ PFU/ml in SM buffer; alone or in combination of 0.25 MIC of Sn) to surfaces of each processed sample (raw chicken fillets, processed nuggets and cheese salad). Subsequently, the pretreated samples were placed in sterile Petri dishes (5 g / each Petri dish), mixed well with a sterile spatula, then kept in fridge (6–8 ° C) for 30 min. Later, 1 ml of the exponentially grown *E. cloacae* E21 (with final concentration of 10^9^ CFU/ml) was added to the treated raw chicken fillets, processed nuggets and cheese salad. Parallel controls were conducted by adding sterile SM buffer or 0.125 mg/ml Sn prior to bacterial addition. The antibacterial activity of the applied bacteriophage (alone or in combination with 0.25 MIC of Sn) was assessed by dispending 1 g of the treated samples in 20 ml SM buffer at 1, 24, 48, 72 and 96 h intervals, mixing them well followed by serial dilution and plating over the selective bismuth sulphite agar for counting *E. cloacae* bacterial cells. Finally, the plates were incubated for 16 – 18 h at 37 ºC and inspected for the positive (black colonies). The antibacterial activity was calculated using the log[N_0_/N_i_], with N_0_ as the initial number of buffer-treated cells and N_i_ as the number of cells counted after treatment with bacteriophage, 0.25 MIC of Sn or the combination of both. The experiment was conducted in fridge conditions (6- 8 ˚C) to simulate the real fillet storage conditions.

### Statistical analysis

Statistical analysis was conducted with GraphPad Prism 9.5 utilizing student's *t*-test for comparing difference between treated and un-treated groups. Statistical significance was considered when *p* < 0.05.

## Results

### Multidrug-resistant *Enterobacter cloacae* from chicken samples

Sixteen *E. cloacae* strains (Table 1) were recovered from the collected chicken fillet samples and confirmed using biochemical and automated Microscan system as described in Sect. 2.1. Antibiotic susceptibility of the identified strains was assessed against a panel of eight routinely prescribed antibiotics for GIT infections. All strains were found resistant to at least three antibiotics (ampicillin, amoxycillin-clavulanic and ceftriaxone; Table 1) with *E. cloacae* EC25 being the most resistant strain (resistant to six out of eight antibiotics; Table 1). These findings highlight food as a potential reservoir harbouring antibiotic resistant *E. cloacae*. Interestingly, all tested strains were sensitive to cefoperazone and cefepime (Table 1), underlining the usefulness of these agents for treatment of food-borne *E. cloacae* infections.

### Bacteriophage isolation, characterization and in vitro antibacterial analysis

Successfully, six bacteriophages (assigned as HK1—HK6) showing clear circular plaques with average sizes ranging from 1- 2 mm in diameter (data not shown) were isolated. Host range analysis of the isolated bacteriophages revealed variable host coverage ranging from one to eight strains out of 16. Bacteriophage HK6 (Enterobacter phage, vB_EclM_HK6) showed the widest spectrum by infecting eight (50%) strains whereas HK3 and HK5 were able to only infect a single strain (6.25%), *E. cloacae* EC38 and EC3 respectively (Table 1). Accordingly, subsequent experiments were conducted using bacteriophage HK6 with its host strain *E. cloacae* EC21.

**Fig. 1:**
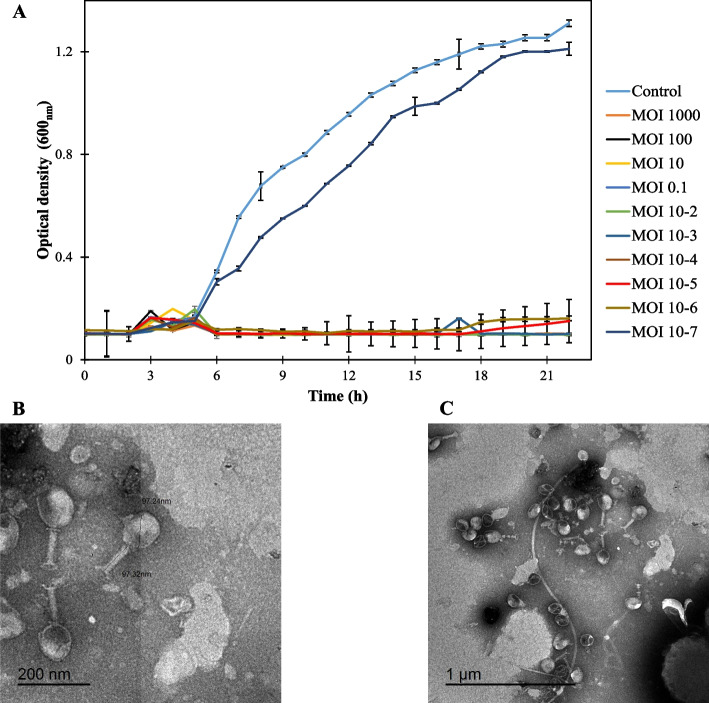
In vitro antibacterial and morphological characterization of HK6. **A** HK6 bacteriolytic activity against exponentially grown *E. cloacae* EC21 mixed at different MOIs (10^3^ – 10^–7^). Each value represents mean ± standard deviation of three replicates. **B** Morphological characterization of HK6 at 200nm scale-bar and **C** 1 µm scale-bar

TEM imaging revealed a classical myovirus morphology (according to the old morpho-based classification by ICTV) with a hexagonal capsid and contractile tail measuring 97.24 and 97.34 nm respectively (Fig. 1B and C). In addition, HK6 was able to completely inhibit bacterial growth irrespective to the used MOI as low as 10^–6^. Nevertheless, lower phage concentration (with final MOI of 10^–7^) showed no bacterial growth inhibition, comparable to the control (Fig. 1A). Interestingly, a slight increase in the optical density was observed for MOIs of 10^–5^ and 10^–6^ suggesting a possible emergence of phage-resistant mutants (Fig. 1C). Mixing phage HK6 with *E. cloacae* EC21 at MOI of 10 resulted in appearance of survivor bacterial colonies with an overall frequency of 5.7 × 10^–4^ ± 3.0 × 10^–4^

### Phage hk6 infection kinetics and stability assessment

**Fig. 2:**
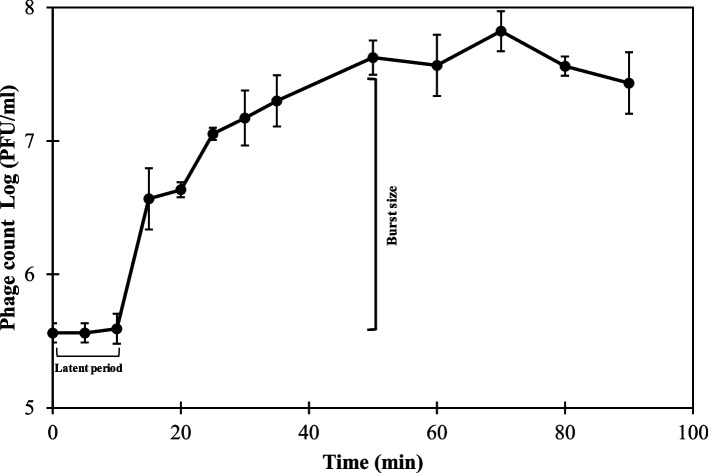
One-step growth curve for HK6 infecting *E. cloacae* EC21. HK6 was mixed with *E. cloacae* EC21 grown (OD 600_nm_) till exponential phase at final MOI of 0.01 and monitored for 90 min**.** Each value represents mean ± standard deviation of three replicates

Phage HK6 revealed a typical growth curve with clear three phases. The phases started with unchanged phage count for at least 10 min (latent period) followed by exponential increase in phage count by 115 ± 44 phage particles (burst size) over a period of 40 min reaching approximately 10^7^ PFU/ml. The curve ended with a plateau till the end of the experiment (90 min; Fig. 2).

**Fig. 3:**
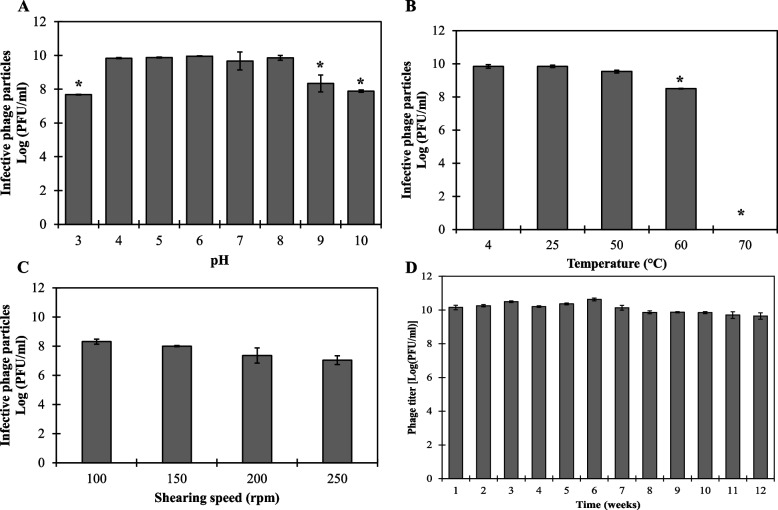
The stability of HK6 regarding different external conditions. **A** pH stability of HK6 diluted in Britton Robinson universal buffer with adjusted pH (3–11) for 1 h at 25˚C. **B** Thermal stability of HK6 (prepared in SM buffer, pH 7.5) after exposure to varying temperatures (4 – 70 °C) for 1 h. **C** HK6 stability to different shearing speeds (100 – 250 rpm) at 25˚C. **D** Storage stability of HK6 (prepared in SM buffer, pH 7.5) over a period of 12 weeks at fridge. Results expressed as the mean infective HK6 [Log(PFU/ml)] of a three replicates ± standard deviation. Student t test was conducted to compare significance between mean values using temperature of 4°C (**A**), pH 7 (**B**), 100 rpm (**C**), and initiation log phage count of 10 (**D**) as references. Asterisks represent statistical significance to the used reference (*p < 0.05)

HK6 retained its full infectivity over a wide pH range (3 – 10) without significant count reduction (*p* > 0.05; Fig. 3A). Nevertheless, a 2.27 log-unit reduction (*p* < 0.05) was observed by subjecting HK6 to pH 3 for 1 h. Similarly, exposing HK6 to a highly alkaline pH values of 9 and 10 resulted in 1.61 and 2.07 log-unit reductions respectively (*p* > 0.05; Fig. 3A). Thermally, HK6 showed a typical mesophilic profile being un-affected up to 50 ºC for 1 h. However, increasing temperature up to 60 ºC caused 1.03 log-unit reduction (*p* < 0.05) in phage count (Fig. 3B). While elevating temperature up to 70 ºC completely abolished HK6 infectivity (*p* < 0.05; Fig. 3B). Mechanically, HK6 showed a relatively high stability towards increasing shaking speed up to 250 rpm at room temperature without significant reduction in overall phage titre within the tested speed (*p* > 0.05; Fig. 3C). Storage stability at 4 ºC in SM buffer was assessed over a period of 12 weeks. Favourably, the phage showed fixed titre (*p* > 0.05) over the tested period in fridge (*p* > 0.05; Fig. 3D).

#### In silico analysis of the phage genome

**Fig. 4:**
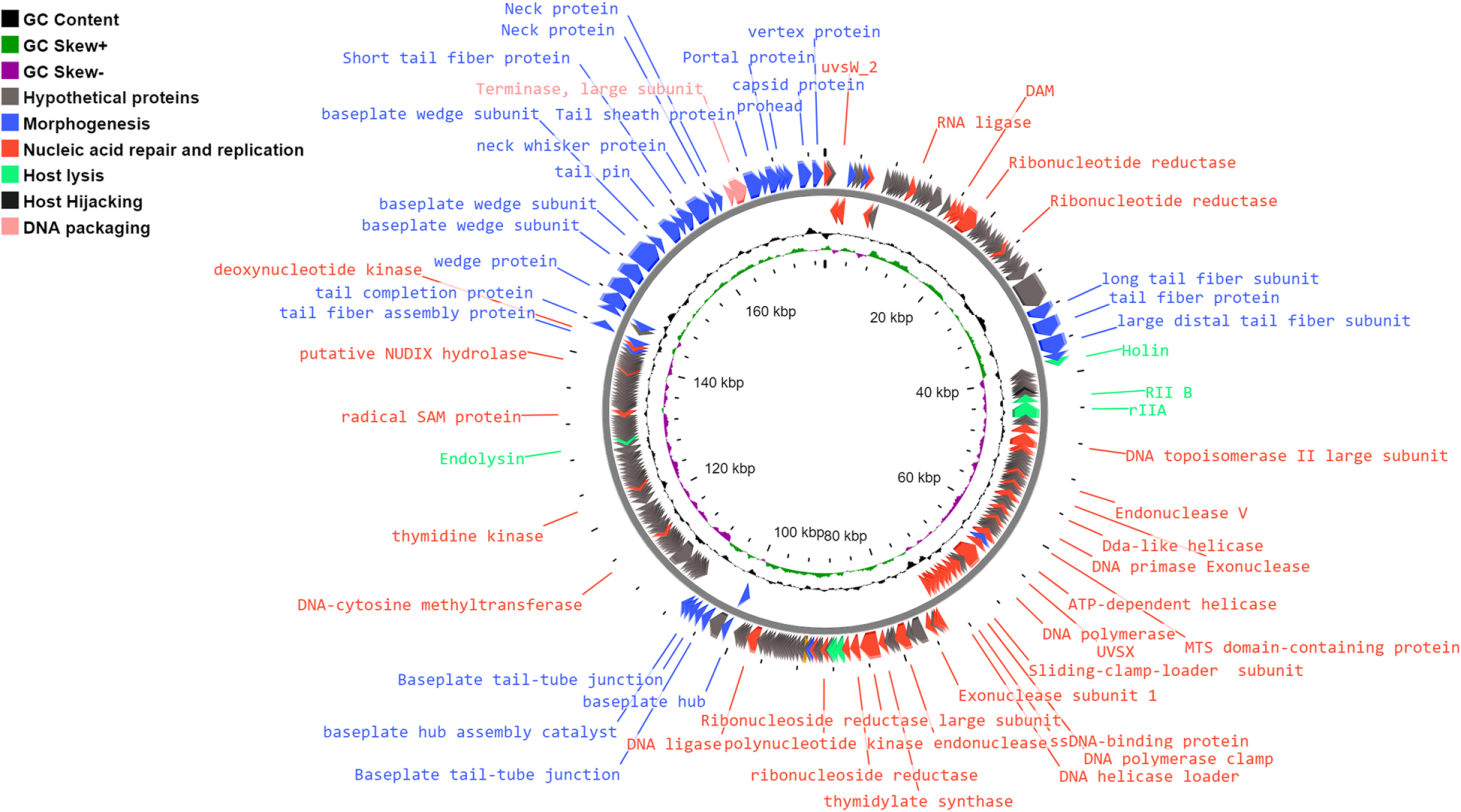
CG viewer circular map of HK6 genome. The outermost ring represents the annotated coding regions with colours indicating their predicted functions as indicated in colour key (left-handed corner). Hypothetical proteins’ labels (grey arrows) were omitted due to size limitations. The second and third rings, from outside, represent GC content and GC skew respectively

In silico analysis of HK6 genome revealed a linear double-stranded DNA genome with an overall size and GC content of 177,845 bp and 44.9% respectively (Accession number PP337149). Only one tRNA gene coding for tRNA_Gly_ and covering 76 nucleotides was detected between open reading frames (*orf*139 – *orf*137; Fig. 4 & Table S1 in supplementary material). RAST annotation and BLASTx analysis have annotated 279 coding sequences on both standard and reverse strands (Fig. 4 & Table S1 in the supplementary material). Functional analysis, using CDD, HHpred and InterPro Scan servers, of the coding sequences have predicted functions of 102 (36.6%) ORFs. Whereas the remaining 177 (63.4%) coding sequences were assigned as hypothetical proteins of unknown function. Functional proteins were categorized into five clearly differentiated groups: Nucleic acid repair and replication, morphogenesis, host lysis, host hijacking and DNA packaging genes (Fig. 4; see Table S1 in the supplemental material).

BLASTn analysis of HK6 genome showed 100 bacteriophages (https://blast.ncbi.nlm.nih.gov/Blast.cgi, accessed on 10th February 2024) with similarity levels ranging from 82 to 98.5%. HK6 showed the highest similarity with Enterobacter phage EBPL (MT341500.1) with identity of 98.5 (coverage, 98%). Moreover, comparable similarities were observed with other phages infecting *Cronobacter spp., Citrobacter spp. and Klebsiella spp*. (Table S2, in the supplemental material). Interestingly, all similar bacteriophages belong to *Straboviridae* (myoviruses) family under class of *Caudoviricetes* (tailed phages), supporting morphological findings observed by TEM. Intergenomic sequence analysis using VIRIDIC showed the highest relatedness to Enterobacter phage EBPL (95.8%, MT341500.1) and Enterobacter phage EC-W1 (95.1%, MN508621.2), exceeding the similarity cut-off value (95%) for species demarcation. This suggests that Enterobacter phage HK6 belongs to the same species as Enterobacter phage and Enterobacter phage EC-W1 (*Pseudotevenvirus leb*). This was further supported by proteomic tree that was constructed using ViPTree (Figure S2).

The lytic nature of HK6 phage was screened using AI-based online tools. Comprehensive Antibiotic Resistance Database (CARD) and Resfinder detected no virulence or antibiotics resistance genes throughout the full genome. In addition, PhageAI analysis revealed the lytic nature of the phage which is further confirmed by absence of lysogenic cycle-related genes. Given, the lytic nature of HK6 together with absence of any potential harmful genes, refuting possible disseminating resistance or virulence genes amongst bacterial population.

### Ex vivo assessment of HK6 safety

**Fig. 5:**
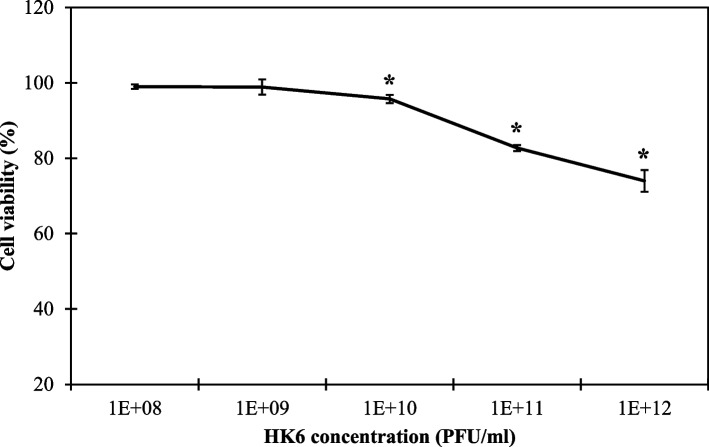
Cytotoxicity of HK6 against oral epithelial human cell line. Cells with final density of 10^4^ cell/well were exposed to ascending HK6 concentrations (10^8^ – 10 ^12^) and mixed with MTT (2.5 µg/ml). Colorimetric changes with respect to (untreated wells) were used for calculating cell viability as percentage. Each value represents the average of three replicates ± standard deviation. Student’s-t test was conducted to detect statical different using untreated cells as reference. Asterisks (*) represent statistical difference from untreated cells

Since HK6 is proposed to be exploited as food additive, its safety was further assessed ex vivo against oral epithelial cells using MTT assay. HK6 showed acceptable safety profile with showing no abrupt change in cell viability with the tested concentrations. For instance, phage concentrations up to 10^9^ PFU/ml displayed no significant cell viability cell change (*p* > 0.05). However, higher tested phage concentrations (10^10^—10^12^ PFU/ml) showed minor changes in cell viability up to 26 ± 6.0 (*p* < 0.05) without detectable IC_50_ nor IC_90._ (Fig. 5). These findings corroborate the evidence acquired from in silico analysis about the safe application of HK 6.

### Biocontrolling *Enterobacter cloacae* with HK6 alone and with sodium nitrite

**Fig. 6:**
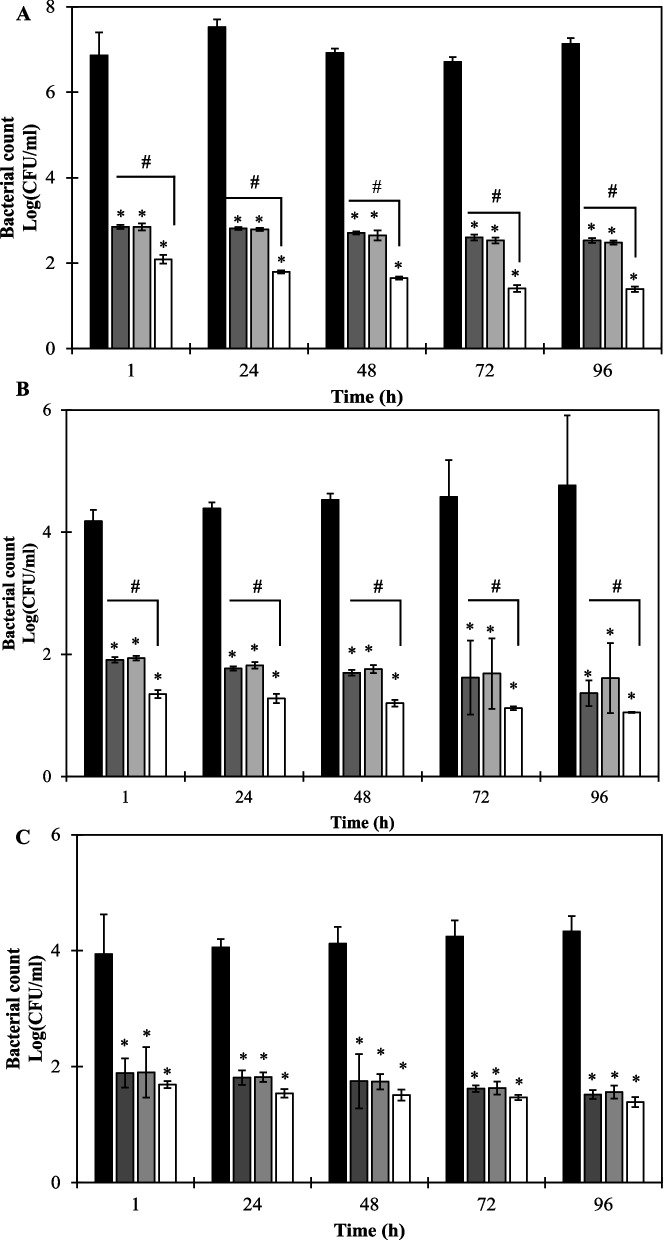
The preservative capacity of HK6, sodium nitrite and combination of both in (**A**) raw chicken fillets, (**B**) chicken nuggets and (**C**) cheese salad. Food products were pretreated with HK6 (10^12^ PFU/ml; dark grey bars), Sn (0.1mg/ml; light grey bars) or combination of both (white bars) then challenged with 1 ml of the exponentially grown *E. cloacae* EC21 (final concentration of 10.^9^ CFU/ml), incubated then sampled at different time points for bacterial counts. The results expressed as log (CFU/ml) of bacterial count. Each value represents mean of three independent replicates ± standard deviation. Asterisks represent statistical differences compared to untreated cells (Student’s *t*-test). Hashtags (#) represent statistical differences in remaining bacterial counts [log (CFU/ml)] in food products treated with HK6 and Sn combination compared to those treated with individual agents separately. The experiment was conducted in refrigerated condition (6–8 ˚C)

HK6 pretreatment showed a fast, and product-dependant bacterial count reduction with the most noticeable effect in case of chicken fillet samples. In addition, the observed activity was less time-dependant with considerable bacterial count reduction within 1 h. For instance, HK6 pretreatment of raw chicken fillets caused a 4.01 log-units bacterial count reduction within 1 h. This effect was slightly improved with additional 0.7 log-unit reduction after 96 h postinfection (*p* < 0.05; Fig. 6A). The preservative capacity of HK6 appeared lower in case of processed chicken nuggets and cheese salad under the same condition. For example, 2.27 and 2.05 log-unit reductions were observed after 1 h HK6 pretreatment of processed chicken nuggets and cheese salad respectively. An additional 1.13 and 0.7 log-unit reductions in bacterial count were detected at 96 h postinfection (Fig. 6B, C). The lower activity of HK6 in both processed and acidic food products suggest the effect of food composition on the overall HK6 activity.

In addition, we investigated whether sodium nitrite could enhance the preservative activity of HK6 in different food matrices. For this, a separate MIC experiment was conducted for Sn using *E. cloacae* EC21, then evaluated the preservative capacity of 0.25 MIC alone and in combination with HK6 in the same food products. Alone, Sn showed MIC of 0.5 mg/ml against *E. cloacae* EC21, thus we used a concentration of 0.1 mg/ml for subsequent food experiments. Using 0.1 mg/ml of Sn showed a comparable preservative capacity with the maximum activity in raw chicken fillets (up to 4.6 log unit reduction; Fig. 6A). However, combining HK6 (10^12^ PFU/ml) and Sn (final concentration of 0.1 mg/ml) resulted in a significant improvement (*p* < 0.5) in bacterial count reduction in case of chicken fillets and processed nuggets by additional 1.03 and 0.56 log units respectively (Fig. 6A-B). On the other hand, this combination was additive in case of cheese-salad (Fig. 6C).

## Discussion

Our initial microbiological analysis of thirty fresh chicken samples revealed high prevalence (16/30 samples, 53.3%) of *E. cloacae* strains. The identified strains show resistance to at least three antibiotics, with other strains displaying broader resistance pattern, EC25, EC29, EC38 (Table 1). Occurrence of multidrug-resistant *E. cloacae* strains within different food matrices has been reported previously. For instance, Edris et al. (2023) identified *E. cloacae*, with a relatively lower prevalence (7/274, 2.5%), upon microbiological analysis of various animal-derived products (*n* = 274) [5]. Another survey, performed in China, reported 13 *E. cloacae* strains harbouring bla_NDM-1_ encoding genes during microbiological analysis [8]. These findings suggest food as a potential reservoir of antibiotic resistance genes, thus posing a hazard, especially when served or stored in healthcare settings hosting immunocompromised patients. Therefore, effective strategies should be explored to minimize this risk.

Using bacteriophages is a promising strategy to control both nosocomial and foodborne pathogens. Here, six bacteriophages were isolated using the chicken-recovered *E. cloacae* strains (Table 1). Enterobacter phage HK6 showed the broadest spectrum, being able to lyse 50% of the tested strains (Table 1). Of note, broad host range is a common feature amongst Enterobacter bacteriophages, with reported bacteriophages covering up to 78% including different *Enterobacter spp*. other than *E. cloacae* [18, 44, 45]. Nevertheless, our work showed bacteriophages, HK3 and HK5, with a specific infectivity against *E. cloacae* EC38 and EC3 respectively (Table 1). Wide host range is a favorable feature, as it allows using the same phage against a larger set of host strains, minimizing the need of formulating phage cocktail comprising numerous individual phages.

HK6 is a strabovirus with short contractile tail and icosahedral capsid (Fig. 1B & C). Interestingly all reported bacteriophages targeting *E. cloacae* have a similar morphotype with comparable head and tail measures [18, 44–46]. This suggests HK6 may be a T4 virus with a lytic productive cycle, a crucial feature required for subsequent application [47]. The lytic nature of HK6 was confirmed using sequential in silico and experimental analysis. Genomic analysis revealed no potential lysogeny, virulence or antibiotic resistance genes. Furthermore, genes responsible for host lysis, [holin (*orf52*), endolysin (*orf213*), spanins (*orfs127, 128, 129*)] were detected (Fig. 4, Table S1 in supplementary material). The lytic cycle of bacteriophage starts with a latent period, where phage number is constant, followed by exponential increasing in phage count, depending on phage burst size [48]. Bacteriophages with short latent period and large burst size are preferential for application. Up on elaboration, HK6 showed a relatively short latent period of 10 min and large burst size of 115 ± 44 PFU/ml (Fig. 2) when compared to other Enterobacter phages [18, 46]. Furthermore, HK6 was able to suppress bacterial growth at MOI as low as 10^–6^ (Fig. 1A). A slight increase in optical density was observed at the end of experiment, suggesting emergence of resistant mutants. This is confirmed by occurrence of phage resistant-mutants with overall frequency of 0.7 × 10^–4^ ± 3.0 × 10^–4^. Phage resistance is a common evolutionary response, arising up on prolonged coincubation via different mechanisms [reviewed in [49]]. Several strategies have been proposed to combat development of phage resistance including using cocktail of phages targeting different molecular sites [50], combination with bacteriocins [51] and combination with chemical agents [52]. Here, we investigated the latter using Sn in different food matrices (discussed below).

Environmental stability is a crucial prerequisite that determines phage suitability for subsequent applications. Enterobacter phage HK6 showed thermal stability up to 60 ˚C without significant count reduction (Fig. 3B). This qualifies its implementation as a biopreservative, whether stored under refrigeration or at room temperature. Moreover, its susceptibility to higher temperature (70 ˚C) allows its easy removal when required. This makes HK6 superior to traditional chemical preservatives, that cannot be removed once added, and may form harmful byproducts upon decay. Enterobacter phage HK6 displayed a wide range of pH stability (3 – 10; Fig. 3A), making it a suitable additive in case of acidic, neutral or alkaline food products. Moreover, its stability under increasing shearing stress (up to 250 rpm), suggests its potential applicability during different food production stages that require agitation e.g., cheese and yogurt production.

Food matrices represent a challenging environment that may potentially interfere with the optimum phage interaction with its bacterial host. Many factors are reported to hamper phage activity in food including food ingredients (e.g., organic acids, fatty acids, tannins) [53], low water content, and media consistency [13]. HK6 showed a product-dependant activity when evaluated in different food matrices. The highest activity was observed in raw chicken samples with overall bacterial count reduction of 4.01 logs after just 1 h of contamination. Whereas a relatively lower activity was observed in processed chicken nuggets and cheese salad, with a bacterial count reduction of 2.27 and 2.05 logs, respectively (Fig. 6). This variation could be explained by the different natures and matrix composition of the tested samples. Chicken nuggets have a higher fat content that may enclose bacterial cells making them unavailable for interaction with phage. Moreover, low water content of chicken nuggets may reduce diffusion of the optimum HK6 concentration for interaction with its host. The concept of a fat protection of bacterial cells is feasible due to the comparable antibacterial pattern obtained after treating the same food products with Sn. The count of bacterial cells adsorbed by each food sample is another factor that may variate the overall activity of the applied bacteriophage in different food samples. In our case, raw chicken samples displayed a relatively higher initial bacterial count than the processed samples (Fig. 6). The higher bacterial count offers bacteriophage an opportunity to perform much more infection cycles, resulting in higher phage number and hence more lysed bacteria (higher antibacterial activity). Despite its superior activity in vitro, HK6 was not able to completely reduce bacterial count in all tested food matrices even after 96 h (Fig. 6). This observation was also reported in several studies validating phage applicability in food materials (reviewed in [53]. Plausible explanation is the development of phage-resistant mutants which is consistent with our in vitro analysis. Nonetheless, other factors including insufficient phage concentrations or unfavourable phage-bacterial interactions (fridge) are also possible, demanding further in-depth investigation.

Bacteriophages are safe and green agents suitable for food applications. Therefore, they were granted Generally Recognized As Safe (GRAS) status by the FDA/USDA for application on various food types. Nevertheless, we conducted an ex vivo safety evaluation for HK6 using human normal oral epithelial cell line (Fig. 5). The tested phage concentration up to 10^12^ PFU/ml displayed a minor cell viability change of 26% suggesting HK6 as a safe candidate for oral consumption. The safety of phage application is mainly attributed to its specific interaction with its target host without disturbing the surrounding environment. Moreover, its thermal sensitivity to cooking temperature also helps in its complete removal prior to consumption, making it an ideal preservative.

Instead of considering phage as alternative for chemical preservative, phage may be combined with conventional preservative. This could be attractive because this combination could result in reducing reliance on the chemical preservative and hence its possible adverse effect. Sn is a well-known chemical preservative used in meat industry. Nonetheless, its serious side effect may limit its safe use as food additive. Combinatorial pretreatment of raw chicken fillets with HK6 (10^12^ PFU/ml) with 0.1 mg/ml Sn showed an additional 1.01 log reduction in bacterial count after 1 h (Fig. 6A). This may be explained by the possible synergistic action on the evolved phage-resistant mutants. However, failure of complete eradication underlines contribution of other factors related to food matrix. This is further reinforced by the different responses observed in chicken nuggets and cheese salad (Fig. 6).

## Conclusion

Occurrence of *E cloacae* within food products alarms for an underestimated route of antibiotic resistance dissemination. In the current study, HK6 showed acceptable antibacterial, kinetic and genomic features making it suitable for food applications. Its limited cytotoxicity a long with its ability to control *E. cloacae* EC21 within different food matrices suggest it a suitable alternative or at least supplement to Sn to reduce the risk of dissemination of this pathogen.

## Supplementary Information

Supplementary Material 1.

## Data Availability

All data generated or analyzed during this study are included in this published article. The nucleotide sequence of Enterobacter phage vB_EclM_HK6 has been deposited in the NCBI GenBank database (accession number PP337149).

## Abbreviations

Sn: Sodium nitrite
LB: Lysogeny broth
S: Second
PFU: Plaque forming unit
CFU: Colony forming unit
MOI: Multiplicity of infection
CDD: Conserved Domain Database
DMEM-F12: Dulbecco’s Modified Eagle Medium/Nutrient Mixture F-12
VIRIDIC: Virus Intergenomic Distance Calculator
IC50: Inhibitory concentration 50
IC90: Inhibitory concentration 90
MIC: Minimum inhibitory concentration
