# Supplementary material for: Characterization and genomic analysis of the lytic bacteriophage vB_EclM_HK6 as a potential approach to biocontrol the spread of Enterobacter cloacae contaminating food
Source: BMC Microbiol. 2024 Oct 15;24:408. doi: 10.1186/s12866-024-03541-9 (PMC11477059; doi:10.1186/s12866-024-03541-9)
Supplement: Supplementary file 1 — Supplementary Material 1. [file 12866_2024_3541_MOESM1_ESM.pdf]

Table S1. Annotation of the HK6 genome and functional analysis of the detected *orf*s using BLASTx analysis against non-redundant data base and search for conserved domains . Predicted function was assigned according to the best hit (highest identity and coverage).

orf	Start (nt)	End (nt)	Length (nt)	Start codon	aa	Predicted function	Closest hit (E-value)	Identity% (coverage %)	Accession number	Conserved domain (E-value)
1	157	579	423	ATG	140	UvsY-like recombination mediator	UvsY-like recombination mediator [Cronobacter phage vB_CsaM_GAP161] (1e-85)	100 (99)	YP_006986492.1	UvsY
2	581	763	183	ATG	60	hypothetical protein	hypothetical protein BANACH_15 [Cronobacter phage vB_CsaD_Banach] (1e-35)	100 (98)	UGO54398.1	DUF2685
3	772	909	138	ATG	35	hypothetical protein	hypothetical protein GAP161_215 [Cronobacter phage vB_CsaM_GAP161] (6e-24)	100 (97)	YP_006986494.1	---
4	1370	1140	231	ATG	76	DNA helicase	DNA helicase [Cronobacter phage vB_CsaM_GAP161] (2e-35)	100 (79)	YP_006986495.1	UvsW
5	2879	1380	1500	GTG	499	DNA helicase	DNA helicase [Cronobacter phage vB_CsaM_leB] (0.0)	99.8 (99)	YP_009831241.1	UvsW<br>DEXHc_UvsW<br>SSL2<br>ResIII<br>dnd_restrict_1
6	2951	3610	660	ATG	219	minor head protein inhibitor of protease	minor head protein inhibitor of protease [Cronobacter phage vB_CsaM_leB] (1e-158)	100 (99)	YP_009831242.1	--
7	3680	4027	348	ATG	115	hypothetical protein	hypothetical protein CPT_Margaery228 [Citrobacter phage Margaery] (3e-67)	100 (99)	YP_009195043.1	--
8	4068	4640	573	ATG	190	hypothetical protein	hypothetical protein CPT_Margaery229	100	YP_009195044.	HAD_SAK_2

							[Citrobacter phage Margaery] (4e-139)	(99)	1	
9	4668	5171	504	ATG	167	Hoc-like head decoration	Hoc-like head decoration [Citrobacter phage Margaery] (7e-104)	100 (99)	YP_009195045.1	---
10	5192	5692	501	ATG	166	5'-3' deoxyribonucleotidase	5'-3' deoxyribonucleotidase [Cronobacter phage vB_CsaM_GAP161] (5e-121)	100 (99)	YP_006986501.1	--
11	6193	5726	468	ATG	155	5'-3' deoxyribonucleotidase	5'-3' deoxyribonucleotidase [Cronobacter phage vB_CsaM_GAP161] (4e-109)	100 (99)	YP_006986502.1	HAD_5-3dNT
12	7076	6243	834	ATG	277	hypothetical protein	hypothetical protein HWB00_gp232 [Cronobacter phage vB_CsaM_leB] (0)	100 (99)	YP_009831248.1	--
13	7191	8060	870	ATG	289	hypothetical protein	hypothetical protein ECW1_0026 [Enterobacter phage EC-W1] (0)	98.96 (99)	URP85422.1	--
14	8097	8414	318	ATG	105	hypothetical protein	hypothetical protein GAP161_226 [Cronobacter phage vB_CsaM_GAP161] (3e-72)	100 (99)	YP_006986505.1	--
15	8411	8584	174	ATG	57	hypothetical protein	hypothetical protein GAP161_227 [Cronobacter phage vB_CsaM_GAP161] (2e-32)	100 (98)	YP_006986506.1	--
16	8577	8942	366	ATG	121	hypothetical protein	hypothetical protein BANACH_30 [Cronobacter phage vB_CsaD_Banach] (1e-85)	100 (99)	UGO54413.1	--
17	8939	9265	327	ATG	108	hypothetical protein	hypothetical protein CPT_Margaery238 [Citrobacter phage Margaery] (2e-73)	100 (99)	YP_009195053.1	--
18	9268	9612	345	ATG	114	hypothetical protein	hypothetical protein HWB00_gp238 [Cronobacter phage vB_CsaM_leB]	100 (99)	YP_009831254.1	--

							(4e-76)			
19	9622	10149	528	ATG	175	hypothetical protein	hypothetical protein EBPL_00245 [Enterobacter phage EBPL] (7e-126)	100 (99)	QJI53286.1	--
20	10152	11195	1044	ATG	347	RNA ligase	RNA ligase [Cronobacter phage vB_CsaM_leN] (0)	100 (99)	AOG16651.1	RNA_lig_RNL2 T4_Rnl2_C
21	11185	11766	582	ATG	193	hypothetical protein	hypothetical protein [Cronobacter phage Dev_CS701] (5e-142)	100 (99)	UPW35660.1	--
22	11836	12279	444	ATG	147	hypothetical protein	hypothetical protein ECW1_0035 [Enterobacter phage EC-W1] (1e-104)	100 (99)	URP85431.1	--
23	12367	12618	252	ATG	83	hypothetical protein	hypothetical protein GAP161_235 [Cronobacter phage vB_CsaM_GAP161] (3e-53)	100 (98)	YP_006986514.1	--
24	12628	13092	465	ATG	154	hypothetical protein	hypothetical protein HWB01_gp248 [Cronobacter phage vB_CsaM_leE] (1e-86)	100 (99)	YP_009831544.1	--
25	13116	14549	1434	ATG	477	hypothetical protein	nicotinamide phosphoribosyl transferase [Cronobacter phage vB_CsaM_leB] (0)	100 (99)	YP_009831261.1	NadV PBEF_like DUF5598
26	14530	14838	309	GTG	102	hypothetical protein	hypothetical protein CPT_Margaery247 [Citrobacter phage Margaery] (1e-65)	98.08 (99)	YP_009195062.1	----
27	14835	15674	840	ATG	479	DNA methylase	DNA methylase [Cronobacter phage vB_CsaM_leN] (0)	100 (99)	AOG16658.1	N6_N4_Mtase PRK11524 YhdJ
28	15671	16522	852	ATG	283	DNA adenine methylase	DNA adenine methylase [Cronobacter phage JC03] (0)	100 (99)	QVW27292.1	dam Dam MethyltransfD12

										PRK10904
29	16529	16798	270	ATG	89	thioredoxin	thioredoxin [Citrobacter phage Margaery] (8e-60)	100 (98)	YP_009195065.1	GRX_GRXb_1_3_like GrxC GlrX-dom PRK10638
30	16831	17313	483	ATG	160	recombination endonuclease	recombination endonuclease [Cronobacter phage vB_CsaM_leN] (6e-116)	100 (99)	AOG16661.1	49 Endonuclease_7
31	17310	19439	2130	ATG	709	ribonucleotide reductase of class III (anaerobic), large subunit	ribonucleotide reductase of class III (anaerobic), large subunit [Cronobacter phage Dev_CS701] (0)	99.01 (99)	UPW35670.1	PRK09263 NrdD NrdD NRDD RNR_III
32	19510	19992	483	ATG	160	hypothetical protein	hypothetical protein RB16p245 [Escherichia phage RB16] (4e-114)	99.38% (99)	YP_003858545.1	--
33	19989	20231	243	ATG	80	hypothetical protein	hypothetical protein EBPL_00260 [Enterobacter phage EBPL] (2e-52)	100 (98)	QJI53301.1	--
34	20228	20728	501	ATG	166	hypothetical protein	hypothetical protein N_260 [Cronobacter phage vB_CsaM_leN] (9e-118)	100 (99)	AOG16665.1	--
35	20738	21334	597	ATG	198	hypothetical protein	hypothetical protein GAP161_244 [Cronobacter phage vB_CsaM_GAP161] (3e-124)	100 (99)	YP_006986523.1	--
36	21364	22311	948	ATG	315	hypothetical protein	hypothetical protein CPT_Maroon_254 [Citrobacter phage Maroon] (0)	99.68% (99)	AYJ73117.1	---
37	22305	22589	285	ATG	94	hypothetical protein	hypothetical protein HWB01_gp260 [Cronobacter phage vB_CsaM_leE] (3e-63)	100 (98)	YP_009831556.1	--

38	22635	22829	195	ATG	64	hypothetical protein	hypothetical protein RB16p248 [Escherichia phage RB16] (7e-38)	100 (98)	YP_003858548.1	--
39	22826	23356	531	ATG	176	hypothetical protein	hypothetical protein CPT_Margaery260 [Citrobacter phage Margaery] (4e-123)	99.43% (99)	YP_009195075.1	--
40	23356	23847	492	ATG	163	ribonucleotide reductase of class III (anaerobic), activating protein	ribonucleotide reductase of class III (anaerobic), activating protein [Enterobacter phage EC-F2] (1e-117)	100 (99)	URP86279.1	NrdG<br>Fer4_12<br>NrdG<br>NrdG<br>HPDL_rSAM_activ<br>Radical_SAM
41	23904	24065	162	ATG	53	hypothetical protein	hypothetical protein HWB00_gp260 [Cronobacter phage vB_CsaM_leB] (2e-28)	100 (98)	YP_009831276.1	--
42	24070	25020	951	ATG	316	hypothetical protein	hypothetical protein EBPL_00268 [Enterobacter phage EBPL] (2e-173)	98.30 (92)	QJI53309.1	-
43	25069	25803	735	ATG	244	hypothetical protein	hypothetical protein HWB01_gp266 [Cronobacter phage vB_CsaM_leE] (6e-165)	98.36 (99)	YP_009831562.1	-
44	25763	26041	279	ATG	92	NrdH glutaredoxin	NrdH glutaredoxin [Escherichia phage RB16] (4e-60)	100 (98)	YP_003858553.1	Glutaredoxin (GRX)<br>Arsenate reductase and related proteins
45	26042	26380	339	ATG	112	hypothetical protein	hypothetical protein CPT_Margaery265 [Citrobacter phage Margaery] (2e-67)	100 (89)	YP_009195080.1	--
46	26377	27804	1428	ATG	475	hypothetical protein	hypothetical protein HWB00_gp265 [Cronobacter phage vB_CsaM_leB] (0)	99.79 (99)	YP_009831281.1	--
47	27861	31520	3660	ATG	121	hypothetical protein	hypothetical protein EBPL_00273	99.51	QJI53314.1	34

					9		[Enterobacter phage EBPL] (0)	(99)		34 Slr4-like
48	31526	33121	1596	ATG	531	long tail fiber proximal subunit	long tail fiber proximal subunit [Enterobacter phage EBPL] (0)	99.62 (99)	QJI53315.1	PANDER_like ILEI
49	33172	35340	2169	ATG	722	tail fiber protein	tail fiber protein [Enterobacter phage EBPL] (0)	96.54 (99)	QJI53316.1	Peptidase_S74 34 choice_anch_A
50	35403	37634	2232	ATG	743	large distal tail fiber subunit	large distal tail fiber subunit [Cronobacter phage vB_CsaM_Invicta] (0)	97.85 (99)	UGV22810.1	34 Peptidase_S74 auto_AIDA-I
51	37661	38179	519	ATG	172	tail fiber assembly protein	tail fiber assembly protein [Klebsiella phage Kp_GWPR59] (1e-106)	98.26 (99)	WAW44527.1	GP38
52	38232	38876	645	ATG	214	holin	holin [Enterobacter phage ENC19] (4e-156)	99.53 (99)	UIW12707.1	Phage_holin_T
53	39433	39131	303	ATG	100	hypothetical protein	hypothetical protein AKFOPBLP_00048 [Cronobacter phage JC03] (2e-67)	100 (99)	QVW27270.1	--
54	40230	39430	801	ATG	266	hypothetical protein	hypothetical protein [Enterobacter phage PF-CE2] (0)	98.87 (99)	QTJ24441.1	--
55	40535	40308	228	ATG	75	hypothetical protein	hypothetical protein HWB00_gp275 [Cronobacter phage vB_CsaM_leB] (3e-49)	100 (98)	YP_009831291.1	--
56	40861	40532	330	ATG	109	hypothetical protein	hypothetical protein CPT_Margaery276 [Citrobacter phage Margaery] (5e-72)	95.41 (99)	YP_009195091.1	--
57	40970	40836	135	ATG	44	hypothetical protein	hypothetical protein RB16p266 [Escherichia phage RB16]	100 (97)	YP_003858566.1	--

							(2e-22)			
58	41266	41018	249	ATG	82	hypothetical protein	hypothetical protein [Enterobacter phage PF-CE2] (3e-49)	100 (98)	QTJ24445.1	Smc
59	41718	41281	438	ATG	145	Ndd-like nucleoid disruption protein	Ndd-like nucleoid disruption protein [Cronobacter phage vB_CsaM_leB] (8e-102)	100 (99)	YP_009831295.1	--
60	42106	41693	414	ATG	137	hypothetical protein	hypothetical protein [Enterobacter phage PF-CE2] (6e-94)	100 (99)	QTJ24447.1	--
61	43051	42152	900	ATG	299	RIIB lysis inhibitor	RIIB lysis inhibitor [Cronobacter phage vB_CsaM_leB] (0)	100 (99)	YP_009831017.1	--
62	45300	43051	2250	ATG	749	RIIA protein	putative rIIA protein [Cronobacter phage vB_CsaD_Banach] (0)	99.73 (99)	UGO54460.1	--
63	45547	45311	237	ATG	78	hypothetical protein	hypothetical protein EBPL_00010 [Enterobacter phage EBPL] (1e-49)	100 (98)	QJI53053.1	--
64	46083	45544	540	ATG	179	hypothetical protein	hypothetical protein HWB00_gp004 [Cronobacter phage vB_CsaM_leB] (2e-131)	100 (99)	YP_009831020.1	
65	47445	46156	1290	ATG	429	DNA gyrase subunit A	DNA gyrase subunit A [Enterobacter phage PF-CE2] (0)	100 (99)	QTJ24178.1	DNA_topoisoIV<br>TOP4c<br>TOP4c<br>GyrA<br>gyrA<br>GyrA_w_intein<br>GyrA_w_intein
66	49337	47442	1896	ATG	631	DNA topoisomerase II large subunit	DNA topoisomerase II large subunit [Cronobacter phage vB_CsaM_leB]	99.84 (99)	YP_009831022.1	TOP2c

							(0)			GyrB gyrB TOPRIM_TopoIIA_like TOPRIM_C
67	49570	49406	165	ATG	54	FmdB-like transcriptional regulator	FmdB-like transcriptional regulator [Cronobacter phage vB_CsaM_GAP161] (2e-34)	100 (98)	YP_006986282.1	COG2331 CxxC_CXXC_SSSS
68	50001	49570	432	ATG	143	putative transcriptional regulator	putative transcriptional regulator [Cronobacter phage vB_CsaM_SemperBestia] (4e-112)	99.3 (99)	QPX76360.1	--
69	50618	50022	597	ATG	198	hypothetical protein	hypothetical protein [Klebsiella phage Kp_GWPR59] (2e-158)	100 (99)	WAW44507.1	---
70	50904	50605	300	ATG	99	hypothetical protein	hypothetical protein CPT_Margaery10 [Citrobacter phage Margaery] (4e-74)	100 (99)	YP_009194825.1	--
71	51491	50901	591	ATG G	196	hypothetical protein	hypothetical protein HWB01_gp011 [Cronobacter phage vB_CsaM_leE] (1e-157)	100 (99)	YP_009831307.1	----
72	51855	51523	333	ATG	110	hypothetical protein	hypothetical protein EBPL_00019 [Enterobacter phage EBPL] (1e-83)	100 (99)	QJI53062.1	--
73	52077	51925	153	ATG	50	hypothetical protein	hypothetical protein HWB00_gp013 [Cronobacter phage vB_CsaM_leB] (4e-31)	100 (98)	YP_009831029.1	--
74	52286	52074	213	ATG	70	hypothetical protein	hypothetical protein [Enterobacter phage vB_EcRAM-01] (4e-21)	100 (98)	UJB55130.1	--
75	52572	52369	204	ATG	67	hypothetical protein	hypothetical protein HWB00_gp015 [Cronobacter phage vB_CsaM_leB]	100 (98)	YP_009831031.1	--

							(2e-44)			
76	52721	52560	162	ATG	53	hypothetical protein	hypothetical protein INVICTA_68 [Cronobacter phage vB_CsaM_Invicta] (8e-30)	100 (98)	UGV22838.1	--
77	53281	52796	486	ATG	161	endonuclease V	endonuclease V N-glycosylase UV repair enzyme [Cronobacter phage vB_CsaM_leB] (3e-125)	100 (99)	YP_009831033.1	--
78	53654	53349	306	ATG	101	hypothetical protein	hypothetical protein GAP161_015 [Cronobacter phage vB_CsaM_GAP161] (1e-73)	100 (99)	YP_006986291.1	--
79	53863	53654	210	ATG	69	hypothetical protein	hypothetical protein NCT2020_0860 [Enterobacter phage vB_EkoM5VN] (4e-48)	100 (98)	BCM29343.1	--
80	54016	53873	144	ATG	47	hypothetical protein	hypothetical protein GAP161_017 [Cronobacter phage vB_CsaM_GAP161] (4e-26)	100 (97)	YP_006986293.1	--
81	54366	54085	282	ATG	93	hypothetical protein	hypothetical protein [Cronobacter phage vB_CsaM_Cronuts] (9e-68)	100 (98)	QPX73389.1	PRK11198 XkdP lipo_LipL71
82	55034	54363	672	ATG	223	exonuclease	exonuclease [Cronobacter phage vB_CsaM_leB] (3e-178)	100 (99)	YP_009831038.1	DexA DUF5051
83	55327	55031	297	ATG	98	hypothetical protein	hypothetical protein CPT_Margaery25 [Citrobacter phage Margaery] (7e-72)	100 (98)	YP_009194840.1	--
84	55638	55324	315	ATG	104	hypothetical protein	hypothetical protein AKFOPBLP_00017 [Cronobacter phage JC03] (1e-73)	97.12 (99)	QVW27239.1	--
85	56996	55665	1332	ATG	443	Dda-like helicase	Dda-like helicase [Cronobacter phage vB_CsaM_leB]	99.77 (99)	YP_009831041.1	recD_rel DEXSc_RecD-like

							(0)			
86	57280	56993	288	ATG	95	hypothetical protein	hypothetical protein HWB00_gp026 [Cronobacter phage vB_CsaM_leB] (2e-69)	100 (98)	YP_009831042.1	--
87	57989	57459	531	ATG	176	hypothetical protein	hypothetical protein [Cronobacter phage vB_CsaM_Cronuts] (1e-139)	100 (99)	QPX73395.1	
88	59022	57997	1026	ATG	341	DNA primase	DNA primase [Citrobacter phage Margaery] (0)	99.41 (99)	YP_009194846.1	
89	59252	59031	222	ATG	73	hypothetical protein	hypothetical protein GAP161_027 [Cronobacter phage vB_CsaM_GAP161] (5e-50)	100 (98)	YP_006986303.1	--
90	59389	59249	141	ATG	46	hypothetical protein	hypothetical protein [Enterobacter phage vB_EcRAM-01] (2e-21)	88.89 (95)	UJB55148.1	--
91	59909	59415	495	ATG	164	hypothetical protein	hypothetical protein phi5_216 [Enterobacter phage phi5] (1e-112)	98.77 (98)	WFD55696.1	Gp23
92	60655	59915	741	ATG	246	MTS domain-containing protein	MTS domain-containing protein [Escherichia phage UPEC03] (0)	99.59 (99)	QUL76959.1	putative methyltransferase type II restriction m6 adenine DNA methyltransferase,
93	61019	60636	384	ATG	127	hypothetical protein	hypothetical protein CPT_Margaery35 [Citrobacter phage Margaery] (3e-94)	100 (99)	YP_009194850.1	--
94	61246	61016	231	ATG	76	hypothetical protein	hypothetical protein [Cronobacter phage vB_CsaM_SemperBestia] (3e-42)	100 (98)	QPX76384.1	--
95	61596	61342	255	ATG	84	hypothetical protein	hypothetical protein CPT_Margaery37 [Citrobacter phage Margaery] (1e-58)	100 (98)	YP_009194852.1	--

96	61886	61677	210	ATG	69	hypothetical protein	hypothetical protein CPT_Margaery38 [Citrobacter phage Margaery] (3e-27)	100 (74)	YP_009194853.1	--
97	63359	61920	1440	ATG	479	DNA helicase	replication and recombination DNA helicase [Citrobacter phage Margaery] (0)	97.7 (99)	YP_009194854.1	helicase
98	63674	63369	306	ATG	101	head vertex assembly chaperone	head vertex assembly chaperone [Citrobacter phage Margaery] (3e-72)	100 (99)	YP_009194855.1	Phage_head_chap
99	64863	63715	1149	ATG	382	Recombination protein	putative recombination protein [Cronobacter phage vB_CsaM_Cronuts] (0)	100 (99)	QPX73406.1	tigrfam_recA
100	67648	64943	2706	ATG	901	DNA polymerase	DNA polymerase [Enterobacter phage vB_EkoM5VN] (0)	99.56 (99)	BCM29363.1	POLBc<br>PolB<br>POLBc<br>pol2<br>DNA_pol_B
101	67958	67641	318	ATG	105	hypothetical protein	hypothetical protein HWB00_gp041 [Cronobacter phage vB_CsaM_leB] (2e-66)	100 (86)	YP_009831057.1	--
102	68389	68027	363	ATG	120	translation repressor	translation repressor [Cronobacter phage vB_CsaM_leB] (2e-90)	100 (99)	YP_009831058.1	RegA<br>Translat_reg
103	68971	68399	573	ATG	190	DNA polymerase clamp loader subunit	DNA polymerase clamp loader subunit [Klebsiella phage Kp_GWPR59] (5e-132)	100 (99)	WAW44474.1	Phage_clamp_A
104	69971	68973	999	ATG	332	clamp loader of DNA polymerase	clamp loader of DNA polymerase [Cronobacter phage vB_CsaM_GAP161] (0)	99.7 (99)	YP_006986318.1	dnaX_nterm<br>RarA<br>RuvB_N<br>RecA-like_PAN_like
105	70701	70036	666	ATG	221	DNA polymerase	DNA polymerase processivity factor	100	YP_006986319.	45

						processivity factor	[Cronobacter phage vB_CsaM_GAP161] (3e-170)	(99)	1	gp45-slide_C
106	71006	70722	285	ATG	94	RpbA RNA polymerase binding protein	RpbA RNA polymerase binding protein [Cronobacter phage vB_CsaM_GAP161] (1e-68)	100 (98)	YP_006986320.1	Phage RNA polymerase binding, RpbA
107	72038	71052	987	ATG	328	putative ssDNA-binding protein	putative ssDNA-binding protein [Cronobacter phage vB_CsaM_Cronuts] (0)	99.04 (94)	QPX73414.1	gp32
108	72707	72042	666	GTG	221	DNA helicase loader	DNA helicase loader [Enterobacter phage EBPL] (3e-167)	97.74 (99)	QJI53095.1	T4_Gp59_N
109	72955	72704	252	ATG	83	late promoter transcriptional regulator	late promoter transcriptional regulator [Citrobacter phage Margaery] (1e-58)	100 (98)	YP_009194866.1	Trans_coact
110	73220	72957	264	ATG	87	transcriptional regulator	transcriptional regulator [Cronobacter phage vB_CsaM_GAP161] (8e-61)	100 (98)	YP_006986324.1	DsbA Phage_DsbA
111	74165	73230	936	ATG	311	putative RnaseH ribonuclease	putative RnaseH ribonuclease [Cronobacter phage vB_CsaM_Cronuts] (0)	100 (99)	QPX73418.1	rnh RNaseH_C PIN_T4-like 53EXOc pola
112	74489	75004	516	ATG	171	RNA polymerase sigma factor	RNA polymerase sigma factor [Cronobacter phage vB_CsaM_GAP161] (2e-123)	100 (99)	YP_006986326.1	RNA polymerase sigma factor;
113	75001	75228	228	ATG	75	RNA polymerase sigma factor	RNA polymerase sigma factor [Cronobacter phage vB_CsaM_GAP161] (2e-123)	100 (99)	YP_006986326.1	RNA polymerase sigma factor
114	75212	75556	345	ATG	114	hypothetical protein	hypothetical protein [Enterobacter phage PF-CE2] (2e-84)	100 (99)	QTJ24225.1	DUF264

115	75583	76602	1020	ATG	339	recombination endonuclease subunit	recombination endonuclease subunit [Cronobacter phage vB_CsaM_leB] ((0)	100 (99)	YP_009831071.1	endonuclease subunit DNA repair exonuclease
116	76595	76885	291	GTG	96	hypothetical protein	hypothetical protein HWB00_gp056 [Cronobacter phage vB_CsaM_leB] (4e-55)	98.96 (98)	YP_009831072.1	--
117	76860	78563	1704	ATG	567	endonuclease	recombination-related endonuclease [Cronobacter phage Dev_CS701] (0)	100 (99)	UPW35479.1	SbcC SMC_prok_B CALCOCO1 ABC_Class2 taxis_HmpF
118	78626	78748	123	TTG	40	hypothetical protein	hypothetical protein HWB00_gp059 [Cronobacter phage vB_CsaM_leB] (5e-22)	97.5 (97)	YP_009831075.1	---
119	78820	80520	1701	ATG	566	hypothetical protein	hypothetical protein BANACH_136 [Cronobacter phage vB_CsaD_Banach] (0)	100 (99)	UGO54519.1	PTZ00100 DnaJ
120	80579	80806	228	ATG	75	Hypothetical protein	hypothetical protein RB16p059 [Escherichia phage RB16] (5e-47)	100 (98)	YP_003858359.1	---
121	80785	81060	276	ATG	91	hypothetical protein	hypothetical protein NCT2020_1270 [Enterobacter phage vB_EkoM5VN] (9e-61)	100 (98)	BCM29384.1	---
122	81057	81638	582	ATG	193	putative dihydrofolate reductase	putative dihydrofolate reductase [Cronobacter phage vB_CsaD_Banach] (6e-152)	97.41 (99)	UGO54522.1	Dihydrofolate reductase
123	81640	82494	855	ATG ATG ATG	284	putative thymidylate synthase	putative thymidylate synthase [Cronobacter phage vB_CsaD_Banach] (0)	99.65 (99)	UGO54523.1	thymidylate synthase
124	82535	84781	2247	ATG	748	ribonucleotide reductase large	ribonucleotide reductase large subunit [Enterobacter phage PF-CE2]	100 (97)	QTJ24236.1	nrdA NrdE_NrdA

						subunit				NrdA<br>Ribonuc_red_lgC<br>RNR_I
125	84820	85995	1176	ATG	391	ribonucleoside-diphosphate reductase	ribonucleoside-diphosphate reductase 1 subunit beta [Enterobacter phage EBPL] (0)	99.74 (99)	QJI53114.1	nrdB<br>NrdF<br>RNRR2<br>Ribonuc_red_sm<br>RNR_1b_NrdF
126	86278	86676	399	ATG	132	endonuclease II	endonuclease II [Cronobacter phage vB_CsaM_leN] (4e-102)	100 (99)	AOG16474.1	denA<br>GIY YIG_EndoII_Hpy188I_like
127	86639	87796	1158	ATG	385	i-spanin	i-spanin [Enterobacter phage vB_EkoM5VN] ((0)	99.74 (99)	BCM29391.1	rnlA<br>RNA_lig_T4_1
128	87793	88119	327	ATG	108	Rz-like spanin	Rz-like spanin [Citrobacter phage Margaery] (3e-77)	100 (99)	YP_009194886.1	--
129	88116	88433	318	ATG	105	O-spanin	O-spanin [Escherichia phage UPEC03] (6e-51)	99.05 (99)	QUL77002.1	--
130	88418	88591	174	ATG	57	hypothetical protein	hypothetical protein GAP161_071 [Cronobacter phage vB_CsaM_GAP161] (8e-36)	100 (98)	YP_006986347.1	--
131	88588	89475	888	ATG	295	putative polynucleotide kinase	putative polynucleotide kinase [Cronobacter phage vB_CsaM_Cronuts] (0)	100 (99)	QPX73440.1	pseT<br>HAD_PNKP-C<br>selen_PSTK<br>AAA_33<br>Kti12
132	89536	89802	267	ATG	88	hypothetical protein	hypothetical protein CPT_Margaery75 [Citrobacter phage Margaery] (4e-61)	100 (98)	YP_009194890.1	--
133	89799	90053	255	ATG	84	hypothetical protein	hypothetical protein HWB00_gp075	100	YP_009831091.	--

							[Cronobacter phage vB_CsaM_leB] (5e-62)	(98)	1	
134	90053	90583	531	ATG	176	dCMP deoxycytidylate deaminase	dCMP deoxycytidylate deaminase [Escherichia phage UPEC03] (4e-126)	99.43 (99)	QUL77007.1	cd deoxycytidylate_deaminase ComEB ComEB dCMP_cyt_deam_1
135	90588	90932	345	ATG	114	head morphogenesis	head morphogenesis [Cronobacter phage vB_CsaM_GAP161] (1e-80)	100 (99)	YP_006986352.1	--
136	90935	91186	252	ATG	83	hypothetical protein	hypothetical protein [Cronobacter phage Dev_CS701] (1e-140)	98.87 (99)	UPW35592.1	DUF262 COG1479
137	91419	91703	285	ATG	94	hypothetical protein	hypothetical protein EBPL_00182 [Enterobacter phage EBPL] (4e-71)	88.29 (99)	QJI53223.1	--
138	91715	92134	420	ATG	139	hypothetical protein	hypothetical protein GAP161_169 [Cronobacter phage vB_CsaM_GAP161] (3e-60)	100 (98)	YP_006986448.1	--
139	92177	92746	570	ATG	189	hypothetical protein	hypothetical protein HWB01_gp181 [Cronobacter phage vB_CsaM_leE] (7e-69)	100 (99)	YP_009831477.1	--
140	92751	93113	363	ATG	120	hypothetical protein	hypothetical protein GAP161_080 [Cronobacter phage vB_CsaM_GAP161] (2e-81)	99 (99)	QPX73448.1	GrcA
141	93113	93394	282	ATG	93	hypothetical protein	hypothetical protein GAP161_081 [Cronobacter phage vB_CsaM_GAP161] (5e-61)	100 (98)	YP_006986357.1	--
142	93515	93715	201	ATG	66	hypothetical protein	hypothetical protein HWB00_gp084 [Cronobacter phage vB_CsaM_leB] (4e-28)	98.48 (98)	YP_009831100.1	--

143	93784	94074	291	ATG	96	hypothetical protein	hypothetical protein CPT_Margaery86 [Citrobacter phage Margaery] (6e-44)	100 (98)	YP_009194901.1	--
144	94077	94307	231	ATG	76	hypothetical protein	hypothetical protein HWB00_gp086 [Cronobacter phage vB_CsaM_leB] (9e-48)	98.68 (98)	YP_009831102.1	--
145	94310	94537	228	ATG	75	hypothetical protein	hypothetical protein [Cronobacter phage vB_CsaM_SemperBestia] (1e-46)	100 (98)	QPX76434.1	--
146	94537	94785	249	ATG GG	82	hypothetical protein	hypothetical protein HWB01_gp090 [Cronobacter phage vB_CsaM_leE] (6e-38)	100 (98)	YP_009831386.1	-
147	94782	95012	231	ATG	76	hypothetical protein	hypothetical protein HWB00_gp089 [Cronobacter phage vB_CsaM_leB] (1e-49)	100 (98)	YP_009831105.1	7,8-didemethyl-8-hydroxy-5-deazariboflavin synthase
148	95012	95173	162	ATG	53	hypothetical protein	hypothetical protein phi5_273 [Enterobacter phage phi5] (2e-30)	98.11 (98)	WFD55753.1	--
149	95170	95325	156	GTG	54	hypothetical protein	hypothetical protein CPT_Margaery91 [Citrobacter phage Margaery] (4e-27)	98.04 (98)	YP_009194906.1	--
150	95404	95844	441	ATG	146	hypothetical protein	hypothetical protein INVICTA_143 [Cronobacter phage vB_CsaM_Invicta] (7e-95)	100 (99)	UGV22910.1	Bacteriophage protein GP30.3;
151	95841	96119	279	ATG	92	hypothetical protein	hypothetical protein HWB01_gp095 [Cronobacter phage vB_CsaM_leE] (6e-59)	97.83 (98)	YP_009831391.1	--
152	96122	96343	222	ATG	73	hypothetical protein	hypothetical protein HWB00_gp096 [Cronobacter phage vB_CsaM_leB] (4e-45)	100 (98)	YP_009831112.1	----
153	96340	96957	618	TTG	205	hypothetical protein	hypothetical protein GAP161_086	99.51	YP_006986363.	hypothetical protein

							[Cronobacter phage vB_CsaM_GAP161] (1e-151)	(99)	1	
154	96935	98458	1524	ATG	507	DNA ligase	DNA ligase [Enterobacter phage vB_EkoM5VN] (0)	99.61 (99)	BCM29417.1	DNA ligase; Provisional ATPase-Plipid
155	98551	98823	273	ATG	90	hypothetical protein	hypothetical protein RB16p088 [Escherichia phage RB16] (3e-57)	100 (98)	YP_003858388.1	--
156	98892	99134	243	ATG	80	hypothetical protein	TPA: hypothetical protein [Caudoviricetes sp.] (4e-53)	100 (98)	DAG47350.1	---
157	99127	99411	285	ATG	94	hypothetical protein	hypothetical protein HWB00_gp101 [Cronobacter phage vB_CsaM_leB] (6e-61)	100 (98)	YP_009831117.1	---
158	99408	99782	375	ATG	124	hypothetical protein	hypothetical protein HWB00_gp102 [Cronobacter phage vB_CsaM_leB] (9e-88)	100 (99)	YP_009831118.1	---
159	100882	99779	1104	ATG	367	baseplate hub	baseplate hub [Citrobacter phage Margaery] (0)	100 (99)	YP_009194918.1	Phage-tail_1
160	100945	101523	579	ATG	192	baseplate distal hub subunit	baseplate distal hub subunit [Enterobacter phage EBPL] (2e-127)	100 (99)	QJI53147.1	Baseplate hub distal subunit
161	101520	103268	1749	ATG	582	hypothetical protein	hypothetical protein ECF1_0171 [Enterobacter phage EC-F1] (0)	99.83 (99)	URP86121.1	--
162	103280	104365	1086	ATG	361	tail-tube assembly protein	tail-tube assembly protein [Citrobacter phage Margaery] (0)	100 (99)	YP_009194921.1	T4_tail_cap
163	104375	105238	864	ATG	287	tail tube	tail tube [Cronobacter phage vB_CsaM_GAP161]	100 (99)	YP_006986374.1	baseplate subunit; Provisional

							(0)			
164	105266	106009	744	ATG	247	baseplate hub assembly catalyst	baseplate hub assembly catalyst [Cronobacter phage vB_CsaM_GAP161] (0)	100 (99)	YP_006986375.1	T4_baseplate
165	106002	106559	558	ATG	185	baseplate hub	baseplate hub [Cronobacter phage vB_CsaM_leB] (1e-132)	100 (99)	YP_009831125.1	T4_baseplate
166	106561	106953	393	ATG	130	baseplate wedge subunit	baseplate wedge subunit [Cronobacter phage vB_CsaM_GAP161] (2e-89)	100 (99)	YP_006986377.1	Gene 25-like lysozyme Phage baseplate assembly protein W
167	107476	106979	498	ATG	165	hypothetical protein	hypothetical protein CPT_Margaery111 [Citrobacter phage Margaery] (7e-116)	100 (99)	YP_009194926.1	--
168	107717	107487	231	ATG	76	hypothetical protein	hypothetical protein [Cronobacter phage vB_CsaM_SemperBestia] (3e-47)	100 (98)	QPX76460.1	--
169	107929	107714	216	ATG	71	hypothetical protein	hypothetical protein HWB00_gp113 [Cronobacter phage vB_CsaM_leB] (2e-41)	100 (98)	YP_009831129.1	--
170	108299	108009	291	ATG	96	hypothetical protein	hypothetical protein HWB00_gp115 [Cronobacter phage vB_CsaM_leB] (9e-64)	100 (98)	YP_009831131.1	---
171	108443	108303	141	ATG	46	hypothetical protein	hypothetical protein GAP161_106A [Cronobacter phage vB_CsaM_GAP161] (7e-24)	100 (97)	YP_006986383.1	--
172	108900	108433	468	ATG	155	hypothetical protein	hypothetical protein CPT_Margaery115 [Citrobacter phage Margaery] (4e-92)	99.35 (99)	YP_009194930.1	--
173	109088	108897	192	ATG	63	hypothetical protein	hypothetical protein CPT_Margaery116 [Citrobacter phage Margaery]	100 (98)	YP_009194931.1	---

							(2e-36)			
174	109711	109085	627	ATG	208	hypothetical protein	hypothetical protein [Enterobacter phage PF-CE2] (8e-142)	100 (99)	QTJ24280.1	--
175	111999	109753	2247	ATG	748	hypothetical protein	hypothetical protein HWB00_gp120 [Cronobacter phage vB_CsaM_leB] (0)	100 (99)	YP_009831136.1	-Secreted protein containing bacterial Ig-like domain and vWFA domain<br>-VWA subgroup: Von Willebrand factor type A (vWA) domain
176	112338	112000	339	ATG	112	hypothetical protein	hypothetical protein [Enterobacter phage PF-CE2] (5e-75)	99.11 (99)	QTJ24282.1	--
177	112850	112338	513	ATG	170	hypothetical protein	hypothetical protein HWB00_gp122 [Cronobacter phage vB_CsaM_leB] (4e-119)	100 (99)	YP_009831138.1	--
178	113080	112847	234	ATG	77	hypothetical protein	hypothetical protein HWB00_gp123 [Cronobacter phage vB_CsaM_leB] (7e-49)	100 (98)	YP_009831139.1	--
179	113319	113077	243	ATG	80	hypothetical protein	hypothetical protein HWB01_gp124 [Cronobacter phage vB_CsaM_leE] (1e-51)	100 (98)	YP_009831420.1	---
180	113588	113316	273	ATG	90	hypothetical protein	hypothetical protein CPT_Margaery123 [Citrobacter phage Margaery] (5e-58)	100 (98)	YP_009194938.1	--
181	113909	113631	279	ATG	92	hypothetical protein	hypothetical protein GAP161_115 [Cronobacter phage vB_CsaM_GAP161] (2e-61)	98.91 (98)	YP_006986393.1	--
182	114166	113951	216	ATG	71	hypothetical protein	hypothetical protein CPT_Margaery126 [Citrobacter phage Margaery] (3e-44)	100 (98)	YP_009194941.1	--

183	114647	114207	441	ATG	146	hypothetical protein	hypothetical protein GAP161_116 [Cronobacter phage vB_CsaM_GAP161] (3e-90)	100 (99)	YP_006986394.1	-
184	115555	114644	912	ATG	303	DNA-cytosine methyltransferase	DNA-cytosine methyltransferase [Enterobacter phage EBPL] (0)	100 (99)	QJI53170.1	DNA-methyltransferase (dcm);<br>C-5 cytosine-specific DNA methylase;<br>Site-specific DNA-cytosine methylase
185	115812	115552	261	ATG	86	hypothetical protein	hypothetical protein GAP161_118 [Cronobacter phage vB_CsaM_GAP161] (5e-54)	100 (98)	YP_006986396.1	---
186	116054	115812	243	ATG	80	hypothetical protein	hypothetical protein HWB00_gp131 [Cronobacter phage vB_CsaM_leB] (6e-51)	100 (98)	YP_009831147.1	--
187	117052	116051	1002	TTG	333	hypothetical protein	nucleotidyltransferase [Cronobacter phage vB_CsaM_leB] (0)	99.7 (98)	YP_009831148.1	
188	117435	117049	387	ATG	128	hypothetical protein	hypothetical protein [Cronobacter phage vB_CsaM_Cronuts] (3e-89)	100 (99)	QPX73485.1	--
189	117683	117432	252	ATG	83	hypothetical protein	hypothetical protein GAP161_122 [Cronobacter phage vB_CsaM_GAP161] (1e-53)	100 (98)	YP_006986400.1	--
190	118237	117680	558	ATG	185	hypothetical protein	Select seq gb|URP85600.1| hypothetical protein ECW1_0206 [Enterobacter phage EC-W1] (6e-131)	97.3 (99)	URP85600.1	--
191	118506	118234	273	ATG	90	hypothetical protein	hypothetical protein GAP161_124 [Cronobacter phage vB_CsaM_GAP161] (2e-60)	100 (98)	YP_006986402.1	--

192	118697	118503	195	ATG	64	hypothetical protein	hypothetical protein GAP161_125 [Cronobacter phage vB_CsaM_GAP161] (3e-37)	100 (98)	YP_006986403.1	---
193	119837	118782	1056	ATG	351	putative bifunctional protein	putative bifunctional protein [Cronobacter phage vB_CsaM_SemperBestia] (0)	96.58 (99)	QPX76486.1	bifunctional nicotinamide-nucleotide adenylyltransferase/Nudix hydroxylase
194	120928	120014	915	ATG	304	putative SPFH domain containing protein	putative SPFH domain containing protein [Cronobacter phage vB_CsaM_Cronuts] (0)	100 (99)	QPX73643.1	SPFH_alloslipin SPFH domain / Band 7 family<br><br>Regulator of protease activity HflC
195	121386	121003	384	ATG	127	hypothetical protein	hypothetical protein [Enterobacter phage PF-CE2] (3e-88)	100 (99)	QTJ24302.1	-DUF5856
196	121681	121397	285	ATG	94	hypothetical protein	hypothetical protein [Enterobacter phage ENC20] (4e-65)	100 (98)	UIW12440.1	--
197	122181	121789	393	ATG	130	hypothetical protein	hypothetical protein EBPL_00143 [Enterobacter phage EBPL] (1e-91)	100 (99)	QJI53184.1	
198	122796	122212	585	TTG	285	thymidine kinase	thymidine kinase [Escherichia phage UPEC03] (5e-143)	99.48 (99)	QUL77066.1	thymidine kinase
199	123004	122789	216	ATG	71	hypothetical protein	hypothetical protein HWB00_gp144 [Cronobacter phage vB_CsaM_leB] (2e-45)	100 (98)	YP_009831160.1	--

200	123284	122997	288	ATG	95	hypothetical protein	hypothetical protein EBPL_00146 [Enterobacter phage EBPL] (3e-45)	98.95 (98)	QJI53187.1	--
201	123772	123281	492	ATG	163	hypothetical protein	hypothetical protein CPT_Margaery144 [Citrobacter phage Margaery] (3e-117)	100 (99)	YP_009194959.1	Macro_Poa1p-like tk.4
202	123860	123762	99	ATG	32	hypothetical protein	hypothetical protein CPT_Margaery145 [Citrobacter phage Margaery] (2e-04)	100 (96)	YP_009194960.1	--
203	124003	123857	147	ATG	48	hypothetical protein	hypothetical protein HWB01_gp146 [Cronobacter phage vB_CsaM_leE] (2e-21)	100 (97)	YP_009831442.1	---
204	124323	124003	321	ATG	106	hypothetical protein	hypothetical protein [Cronobacter phage vB_CsaM_Cronuts] (7e-72)	100 (99)	QPX73502.1	--
205	124944	124333	612	ATG	203	hypothetical protein	hypothetical protein [Cronobacter phage vB_CsaM_Cronuts] (2e-138)	99.51 (99)	QPX73503.1	--
206	125350	124946	405	ATG	134	hypothetical protein	hypothetical protein [Cronobacter phage vB_CsaM_Cronuts] (6e-94)	100 (99)	QPX73504.1	--
207	126065	125418	648	ATG	215	hypothetical protein	hypothetical protein EBPL_00155 [Enterobacter phage EBPL] (7e-158)	100 (99)	QJI53196.1	--
208	126684	126130	555	ATG	184	hypothetical protein	hypothetical protein HWB00_gp154 [Cronobacter phage vB_CsaM_leB] (5e-105)	83.24 (99)	YP_009831170.1	--
209	126851	126681	171	ATG	56	hypothetical protein	hypothetical protein HWB00_gp155 [Cronobacter phage vB_CsaM_leB] (2e-30)	100 (98)	YP_009831171.1	--
210	127053	126859	195	ATG	64	hypothetical protein	hypothetical protein INVICTA_207	98.44	UGV22974.1	--

							[Cronobacter phage vB_CsaM_Invicta] (4e-38)	(98)		
211	128434	127091	1344	ATG	447	hypothetical protein	hypothetical protein ECW1_0229 [Enterobacter phage EC-W1] (0)	100 (99)	URP85623.1	YlaK PhoH PRK10536 PIN_VapC_PhoHL-ATPase
212	128959	128489	471	ATG	156	hypothetical protein	hypothetical protein [Cronobacter phage vB_CsaM_Cronuts] (9e-113)	100 (99)	QPX73511.1	---
213	129364	128969	396	ATG	131	endolysin	endolysin [Cronobacter phage vB_CsaM_GAP161] (3e-90)	100 (99)	YP_006986425.1	peptidase M15 family (pfam 02557)
214	129838	129431	408	ATG	135	hypothetical protein	Hypothetical protein Enterobacter phage ENC7 (9e-78)	94 (99)	UIW11515.1	--
215	130214	129894	321	ATG	106	hypothetical protein	hypothetical protein ECF1_0225 [Enterobacter phage EC-F1] (5e-72)	100 (99)	URP86175.1	--
216	130514	130251	264	ATG	87	hypothetical protein	hypothetical protein KP15_130 [Klebsiella phage KP15] (4e-58)	100 (98)	YP_003580006.2	--
217	130850	130587	264	ATG	87	hypothetical protein	hypothetical protein GAP161_150 [Cronobacter phage vB_CsaM_GAP161] (9e-51)	97.7 (98)	YP_006986429.1	
218	131294	130854	441	ATG	146	hypothetical protein	hypothetical protein [Enterobacter phage ENC19] (3e-103)	99.31 (99)	UIW12626.1	--
219	131470	131291	180	ATG	59	hypothetical protein	hypothetical protein CPT_Margaery162 [Citrobacter phage Margaery] (8e-36)	100 (98)	YP_009194977.1	--
220	131820	131467	354	ATG	117	hypothetical protein	hypothetical protein CPT_Margaery163	100	YP_009194978.	

							[Citrobacter phage Margaery] (1e-80)	(99)	1	
221	132175	131795	381	ATG	126	hypothetical protein	hypothetical protein CPT_Margaery164 [Citrobacter phage Margaery] (1e-72)	97.62 (99)	YP_009194979.1	--
222	132468	132175	294	ATG	97	hypothetical protein	hypothetical protein HWB00_gp168 [Cronobacter phage vB_CsaM_leB] (5e-65)	100 (98)	YP_009831184.1	--
223	133619	132468	1152	ATG	383	putative radical SAM protein	putative radical SAM protein [Cronobacter phage vB_CsaM_leE] (0)	100 (99)	YP_009831462.1	tungsten cofactor oxidoreducase radical SAM maturase
224	133836	133630	207	ATG	68	hypothetical protein	hypothetical protein HWB01_gp167 [Cronobacter phage vB_CsaM_leE] (3e-42)	100 (98)	YP_009831463.1	---
225	134093	133833	261	ATG	86	hypothetical protein	hypothetical protein [Cronobacter phage vB_CsaM_SemperBestia] (2e-55)	100 (98)	QPX76516.1	---
226	134239	134093	147	ATG	48	hypothetical protein	hypothetical protein CPT_Margaery169 [Citrobacter phage Margaery] (1e-25)	100 (97)	YP_009194984.1	--
227	134487	134236	252	ATG	83	hypothetical protein	hypothetical protein RB16p161 [Escherichia phage RB16] (3e-53)	100 (98)	YP_003858461.1	
228	134837	134559	279	ATG	91	hypothetical protein	hypothetical protein HWB01_gp171 [Cronobacter phage vB_CsaM_leE] (7e-50)	98.73 (84)	YP_009831467.1	DUF5417
229	135175	134927	249	ATG	82	hypothetical protein	hypothetical protein EBPL_00176 [Enterobacter phage EBPL] (5e-51)	93.90 (98)	QJI53217.1	--
230	135767	135177	591	ATG	196	hypothetical protein	Select seq gb|QPX73528.1| hypothetical protein [Cronobacter phage	97.96 (99)	QPX73528.1	--

							vB_CsaM_Cronuts] (3e-129)			
231	136180	135764	417	ATG	138	hypothetical protein	hypothetical protein [Enterobacter phage PF-CE2] (2e-97)	97.83 (99)	QTJ24340.1	--
232	136307	136191	117	ATG	38	hypothetical protein	hypothetical protein [Cronobacter phage vB_CsaM_Cronuts] (7e-18)	100 (97)	QPX73530.1	PRK1428 DnaJ_zf COG4700
233	136562	136359	204	ATG	67	hypothetical protein	hypothetical protein [Cronobacter phage vB_CsaM_Cronuts] (1e-41)	100 (98)	QPX73531.1	--
234	136947	136642	306	ATG	101	hypothetical protein	hypothetical protein RB16p167 [Escherichia phage RB16] (7e-60)	91.18 (99)	YP_003858467.1	--
235	137477	136944	534	ATG	177	hypothetical protein	hypothetical protein [Cronobacter phage Dev_CS701] (4e-129)	98.87 (99)	UPW35592.1	DUF262 COG1479
236	137805	137470	336	ATG	111	hypothetical protein	hypothetical protein EBPL_00182 [Enterobacter phage EBPL] (4e-65)	89.09 (97)	QJI53223.1	---
237	138073	137813	261	ATG	86	hypothetical protein	hypothetical protein [Enterobacter phage vB_EcRAM-01] (8e-55)	97.67 (98)	UJB55306.1	---
238	138450	138070	381	ATG	126	hypothetical protein HWB01_gp181	hypothetical protein HWB01_gp181 [Cronobacter phage vB_CsaM_leE] (2e-63)	100 (99)	YP_009831477.1	--
239	138860	138447	414	ATG	137	putative NUDIX hydrolase	putative NUDIX hydrolase [Cronobacter phage vB_CsaD_Banach] (7e-98)	100 (99)	UGO54638.1	Ap4A_hydrolase_plant_like NUDIX MutT PRK00714 nudix_YtkD

240	139099	138860	240	ATG	79	hypothetical protein	hypothetical protein CPT_Maroon_179 [Citrobacter phage Maroon] (2e-38)	100 (98)	AYJ73042.1	---
241	139362	139096	267	ATG	88	hypothetical protein	hypothetical protein [Cronobacter phage vB_CsaM_Cronuts] (8e-57)	100 (98)	QPX73539.1	--
242	139648	139346	303	ATG	100	hypothetical protein	hypothetical protein EBPL_00188 [Enterobacter phage EBPL] (1e-68)	99 (99)	QJI53229.1	--
243	139911	139645	267	ATG	88	hypothetical protein	hypothetical protein HWB01_gp186 [Cronobacter phage vB_CsaM_leE] (5e-46)	100 (98)	YP_009831482.1	--
244	140246	139908	339	ATG	112	hypothetical protein	hypothetical protein HWB01_gp187 [Cronobacter phage vB_CsaM_leE] (6e-78)	100 (99)	YP_009831483.1	--
245	140397	140257	141	ATG	46	hypothetical protein	hypothetical protein [Cronobacter phage vB_CsaM_SemperBestia] (1e-12)	100 (65)	QPX76536.1	--
246	140627	140487	141	ATG	46	hypothetical protein	hypothetical protein CPT_Margaery188 [Citrobacter phage Margaery] (5e-26)	100 (97)	YP_009195003.1	--
247	141233	140694	540	ATG	179	hypothetical protein	hypothetical protein CPT_Margaery189 [Citrobacter phage Margaery] (2e-130)	100 (99)	YP_009195004.1	--
248	141878	141255	624	ATG	207	hypothetical protein	hypothetical protein BANACH_265 [Cronobacter phage vB_CsaD_Banach] (8e-148)	98.55 (99)	UGO54646.1	hypothetical protein; Provisional
249	142206	141937	270	ATG	89	tail fiber assembly protein	tail fiber assembly protein [Cronobacter phage Dev_CS701] (1e-52)	97.75 (98)	UPW35608.1	---
250	142878	142210	669	ATG	222	deoxynucleotide	deoxynucleotide monophosphate kinase	94.29	YP_009195007.	deoxynucleoside

						monophosphate kinase	[Citrobacter phage Margaery] (3e-148)	(94)	1	monophosphate kinase; Provisional
251	143414	142875	540	ATG	179	head-proximal tip of tail tube	head-proximal tip of tail tube [Cronobacter phage vB_CsaM_leE] (3e-130)	100 (99)	YP_009831490.1	tail completion and sheath stabilizer protein
252	143683	144468	786	ATG	261	tail completion protein	tail completion protein [Enterobacter phage PF-CE2] (0)	99.62 (99)	QTJ24359.1	--
253	145358	144504	855	ATG	284	hypothetical protein	hypothetical protein AKFOPBLP_00125 [Cronobacter phage JC03] (4e-179)	99.62 (91)	QVW27347.1	DNA end protector protein;
254	145739	145380	360	ATG	119	head completion protein	head completion protein [Enterobacter phage ENC20] (1e-71)	99.16 (99)	UIW12358.1	head completion protein; Provisional
255	145891	146445	555	ATG	184	head completion protein	head completion protein [Enterobacter phage ENC20] (1e-71)	99.16 (99)	UIW12358.1	head completion protein; Provisional
256	146442	148202	1761	ATG	586	baseplate wedge protein	baseplate wedge protein [Enterobacter phage EBPL] (0)	100 (99)	QJI53243.1	PHA02596 Gp5_OB T4-like_lys RrrD
257	148205	150292	2088	ATG	695	baseplate hub structural protein	Select seq gb|QJI53244.1| baseplate hub structural protein [Enterobacter phage EBPL] (0)	99.86 (99)	QJI53244.1	---
258	150316	152229	1914	ATG	637	baseplate wedge subunit	baseplate wedge subunit [Citrobacter phage Margaery] (0)	99.84 (99)	YP_009195015.1	baseplate wedge subunit;
259	152308	155388	3081	ATG	1026	baseplate wedge subunit	baseplate wedge subunit [Cronobacter phage vB_CsaM_leE] (0)	99.9 (99)	YP_009831498.1	baseplate wedge subunit; Provisional

260	155388	156380	993	ATG	330	baseplate wedge subunit	baseplate wedge subunit [Cronobacter phage Dev_CS701] (0)	100 (99)	UPW35619.1	baseplate wedge subunit; Provisional Bacteriophage T4, Gp8
261	156391	157254	864	ATG	287	baseplate wedge tail fiber protein connector	baseplate wedge tail fiber protein connector [Citrobacter phage Margaery] (0)	100 (99)	YP_009195018.1	baseplate wedge tail fiber connector; Bacteriophage T4 gp9/10-like protein
262	157251	159068	1818	ATG	605	baseplate wedge subunit and tail pin	baseplate wedge subunit and tail pin [Enterobacter phage EC-W1] (0)	99.83 (99)	URP85672.1	aseplate wedge subunit and tail pin; Bacteriophage T4 gp9/10-like protein
263	159069	159731	663	ATG	220	baseplate wedge subunit	baseplate wedge subunit [Citrobacter phage Margaery] (3e-160)	100 (99)	YP_009195020.1	baseplate wedge subunit and tail pin; GP11 baseplate wedge protein
264	159741	161129	1389	ATG	462	tail collar fiber protein	tail collar fiber protein [Cronobacter phage vB_CsaM_leE] (0)	100 (99)	YP_009831503.1	ong tail fiber, proximal subunit Phage Tail Collar Domain; Microcystin-dependent protein
265	161143	163431	2289	ATG	762	neck whisker protein	neck whisker protein [Cronobacter phage vB_CsaM_Invicta] (0)	99.74 (99)	UGV23031.1	fibritin; Fibritin C-terminal region
266	163468	164391	924	ATG	307	neck protein	neck protein [Cronobacter phage vB_CsaM_Invicta] (0)	100(99)	UGV23032.1	neck protein; Provisional
267	164400	165146	747	ATG	248	head closure Hc2	head closure Hc2 [Citrobacter phage Margaery] (2e-170)	100 (99)	YP_009195024.1	neck protein; Provisional\ Virus neck protein
268	165205	166026	822	ATG	273	tail sheath stabilizer	tail sheath stabilizer and completion	100	AOG16619.1	tail sheath stabilizer and

						and completion protein	protein [Cronobacter phage vB_CsaM_leN] (0)	(99)		completion protein T4-like virus Myoviridae tail sheath stabilizer
269	166029	166571	543	ATG	180	small terminase subunit	small terminase subunit [Enterobacter phage PF-CE2] (9e-129)	100 (99)	QTJ24377.1	small terminase protein Terminase DNA packaging enzyme
270	166540	168369	1830	ATG	609	terminase large subunit	terminase large subunit [Cronobacter phage vB_CsaM_leB] (0)	100 (99)	YP_009831227.1	Large terminase protein; Terminase-like family
271	168388	170379	1992	ATG	663	tail sheath	tail sheath [Cronobacter phage vB_CsaM_leB] (0)	99.7 (99)	YP_009831228.1	tail sheath protein
272	170425	170910	486	ATG	161	tail tube protein	tail tube protein [Cronobacter phage vB_CsaM_leB] (9e-117)	100 (99)	YP_009831229.1	tail tube protein
273	170964	172538	1575	ATG	524	putative portal vertex protein	putative portal vertex protein [Cronobacter phage vB_CsaD_Banach] (0)	100 (95)	UGO54389.1	portal vertex protein Bacteriophage T4-like capsid assembly protein
274	172538	172792	255	ATG	84	prohead	prohead [Cronobacter phage vB_CsaM_GAP161] (6e-25)	100 (57)	YP_006986485.1	prohead core protein; Gene product 67
275	172805	173212	408	ATG	135	head scaffolding protein	head scaffolding protein [Citrobacter phage Margaery] (3e-77)	100 (86)	YP_009195032.1	prohead core protein;
276	173215	173874	660	ATG	219	prohead core and protease	prohead core and protease [Cronobacter phage vB_CsaM_GAP161] (2e-156)	100 (99)	YP_006986487.1	prohead core scaffolding protein and protease<br><br>Prohead core protein serine protease;
277	173901	174695	795	ATG	264	head scaffolding protein	head scaffolding protein [Citrobacter phage Margaery]	100 (99)	YP_009195034.1	prohead core protein DNA repair exonuclease

							(3e-180)			SbcCD ATPase subunit<br>Tropomyosin like chromosome segregation protein SMC<br>basic Helix-Loop-Helix-zipper
278	174715	176289	1575	ATG	524	prohead assembly/scaffold protein	prohead assembly/scaffold protein [Enterobacter phage EBPL]<br>(0)	100<br>(99)	QJI53265.1	major capsid protein<br>Major capsid protein Gp23;
279	176383	177681	1299	ATG	423	capsid vertex protein	homing endonuclease [Enterobacter phage EBPL]<br>(0)	100<br>(99)	QJI53267.1	Major capsid protein Gp23;

Table S2: Genomic sequence similarly of Enterobacter phage vB_EclM-HK6 with other phages deposited in NCBI. The listed phages are selected based on BLASTn analysis of Enterobacter phage vB_EclM-HK6 assembled genomic sequence.

Bacteriophage name	Identity (%)	Coverage (%)	Accession
Enterobacter phage EBPL	98.47%	98%	MT341500.1
Cronobacter phage vB_CsaM_leB	98.86%	97%	NC_048645.1
Enterobacter phage vB_EkoM5VN	98.15%	97%	LC589952.1
Citrobacter phage Margaery	97.75%	97%	KT381880.1
Citrobacter phage Margaery	97.75%	97%	NC_028755.1
Citrobacter phage Maroon	97.55%	96%	MH823906.1
Cronobacter phage Dev_CS701	97.52%	97%	ON157416.1
Cronobacter phage vB_CsaM_leN	98.79%	97%	KX431560.1
Cronobacter phage vB_CsaM_SemperBestia	97.19%	95%	MW021756.1
Enterobacter phage EC-W1	97.80%	97%	MN508621.2
Cronobacter phage vB_CsaM_Invicta	97.71%	96%	OL539470.1
Cronobacter phage vB_CsaM_Cronuts	98.46%	93%	MW021751.1
Klebsiella phage iPHaGe-KPN-11i	97.03%	93%	OR637327.1
Cronobacter phage vB_CsaM_leE	98.51%	95%	NC_048646.1
Enterobacter phage PF-CE2	97.58%	96%	MW629017.1
Citrobacter phage vB_CfrM_CfP1	85.58%	84%	KX245890.1
Citrobacter phage vB_CfrM_CfP1	85.58%	84%	NC_031057.1
Citrobacter phage vB_Cfr_Xman	85.55%	85%	MW021749.1
Klebsiella phage Kp_GWPR59	98.71%	95%	OP970827.1
Enterobacter phage EC-F2	97.99%	95%	MN508624.2
Cronobacter phage JC03	94.50%	93%	MW767161.1
Enterobacter phage EC-W2	90.70%	94%	MN508622.2
Cronobacter phage vB_CsaM_GAP161	97.32%	93%	NC_019398.1
Cronobacter phage vB_CsaM_GAP161	97.32%	93%	JN882287.1
Enterobacter phage vB_EcRAM-01	92.47%	95%	OL551674.1
Escherichia phage UPEC03	97.90%	92%	MW250785.1
Cronobacter phage vB_CsaD_Banach	98.68%	94%	OL539471.1
Enterobacter phage EC-F1	97.83%	95%	MN508623.2
Buttiauxella phage vB_ButM_GuL6	85.30%	84%	MT334653.1
Enterobacter phage phi5	97.18%	94%	OQ571799.1
Klebsiella phage iPHaGe-KPN-12i	97.53%	94%	OR637326.1
Enterobacter phage ENC22	92.93%	95%	OL355128.1
Enterobacter phage ENC25	92.93%	95%	OL355127.1
Enterobacter phage ENC7	93.77%	95%	OL355125.1
Enterobacter phage ENC20	92.26%	95%	OL355129.1
Enterobacter phage ENC19	91.12%	93%	OL355131.1
Citrobacter phage KKP_3664	83.51%	32%	OK210075.1
Escherichia phage RB16	88.25%	85%	NC_014467.1
Citrobacter phage Miller	87.85%	85%	NC_025414.1
Citrobacter phage Miller	87.85%	85%	KM236237.1
Citrobacter phage IME-CF2	87.83%	84%	KR869820.1
Citrobacter phage IME-CF2	87.83%	84%	NC_029013.1
Caudoviricetes sp.	95.72%	21%	BK018132.1
Escherichia phage Lw1	87.04%	84%	NC_021344.2
Escherichia phage Lw1	87.04%	84%	KC801932.2
Caudoviricetes sp.	97.36%	18%	BK017862.1

Pseudotevenvirus RB43	86.29%	84%	HE858210.2
Pseudotevenvirus RB43	86.29%	83%	HE981739.1
Escherichia phage RB43	86.28%	84%	NC_007023.1
Caudoviricetes sp.	96.33%	9%	BK018324.1
Enterobacter phage phiEap-3	81.29%	54%	NC_041980.1
Klebsiella phage P52_1	81.25%	58%	OR256021.1
Klebsiella phage vB_KpnM_05F	81.24%	58%	LR746310.1
Klebsiella phage P61_1	81.23%	57%	OR256023.1
Klebsiella phage PSKm2DI	81.63%	57%	MZ707156.1
Klebsiella phage R4_1	81.20%	60%	OR138017.1
Klebsiella phage vB_KoM-Pickle	81.20%	58%	LR881145.1
Klebsiella phage vB_KoM-Liquor	81.18%	57%	LR881143.1
Klebsiella phage vB_KpM-Milk	81.18%	57%	LR881142.1
Klebsiella phage vB_KpnM_VAC13	81.18%	58%	MZ322895.1
Klebsiella phage PMBT1	81.18%	57%	NC_042138.1
Klebsiella phage vB_KoM-MeTiny	81.17%	58%	LR883651.1
Klebsiella phage vB_KpM-KalD	81.13%	56%	LR881140.1
Klebsiella phage KP27	81.54%	58%	NC_020080.1
Klebsiella phage phi_KPN_S3	81.53%	58%	OQ267591.1
Klebsiella phage vB_KpnM_M1	81.53%	58%	MW448170.1
Klebsiella phage R6_1	81.49%	57%	OR138015.1
Klebsiella phage Matisse	81.51%	58%	NC_028750.1
Klebsiella phage P-KP2	81.50%	55%	MT157285.1
Klebsiella phage phiKp_26	81.49%	56%	LC768496.1
Klebsiella phage phiKp_23	81.49%	17%	LC768494.1
Klebsiella phage phiKp_19	81.49%	56%	LC768482.1
Klebsiella phage phiKp_18	81.49%	56%	LC768481.1
Klebsiella phage phiKp_17	81.49%	56%	LC768480.1
Klebsiella phage phiKp_16	81.49%	56%	LC768479.1
Klebsiella phage phiKp_15	81.49%	17%	LC768478.1
Klebsiella phage phiKp_13	81.47%	56%	LC768475.1
Klebsiella phage phiKp_12	81.47%	56%	LC768474.1
Klebsiella phage phiKp_11	81.49%	56%	LC768473.1
Klebsiella phage phiKp_10	81.47%	56%	LC768472.1

Klebsiella phage phiKp_9	81.49%	17%	LC768471.1
Klebsiella phage phiKp_8	81.49%	56%	LC768469.1
Klebsiella phage phiKp_5	81.47%	56%	LC768466.1
Klebsiella phage phiKp_4	81.47%	56%	LC768465.1
Klebsiella phage phiKp_3	81.47%	56%	LC768464.1
Klebsiella phage phiKp_1	81.47%	56%	LC768463.1
Klebsiella phage KP15	81.48%	56%	NC_014036.1
Klebsiella phage CPRSB	81.26%	56%	OM971649.1
Klebsiella phage CPRSA	81.23%	56%	OM971648.1
Escherichia phage phT4A	82.02%	52%	NC_055712.1
Klebsiella phage 150040	81.64%	59%	OP045497.1
Klebsiella phage KpF2	81.61%	59%	OR234023.1
Klebsiella phage PSKm4DII	82.05%	56%	MZ707157.1
Klebsiella phage KP13MC5-1	82.06%	52%	OP617746.1
Klebsiella phage vB_KpM-SoFaint	82.00%	58%	LR881141.1
Klebsiella phage vB_KpM-Mild	81.99%	57%	LR881147.1
Klebsiella phage mtp7	81.95%	56%	OX335407.1
Klebsiella phage R2_1	82.09%	54%	OR138016.1
Klebsiella phage KMI7	81.83%	39%	MN101221.1
Klebsiella phage KMI9	82.20%	54%	MN101223.1

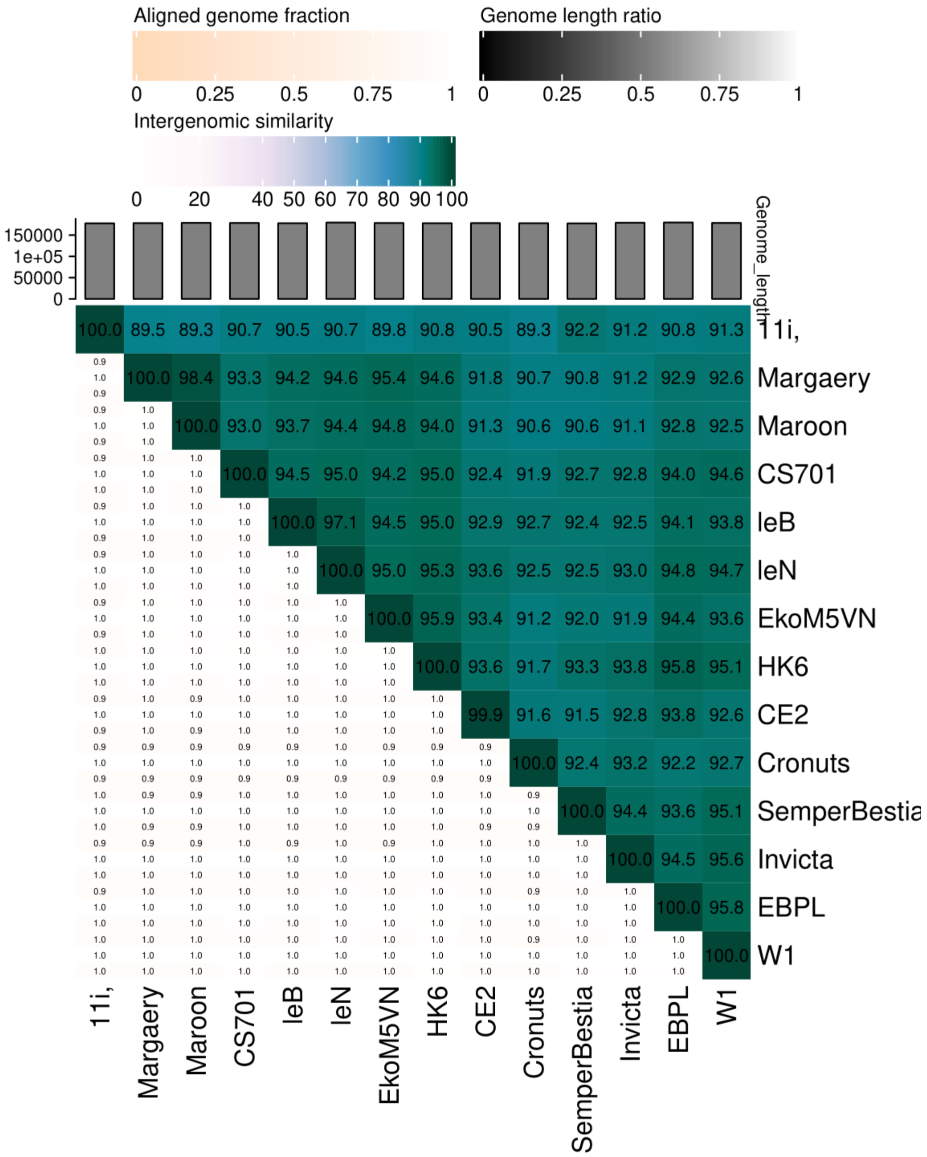

Figure S1: Heatmap of Intergenomic Similarity of Enterobacter phage vB_EclM-HK6 with genomic-close Enterobacter phages obtained using VIRIDIC.

E

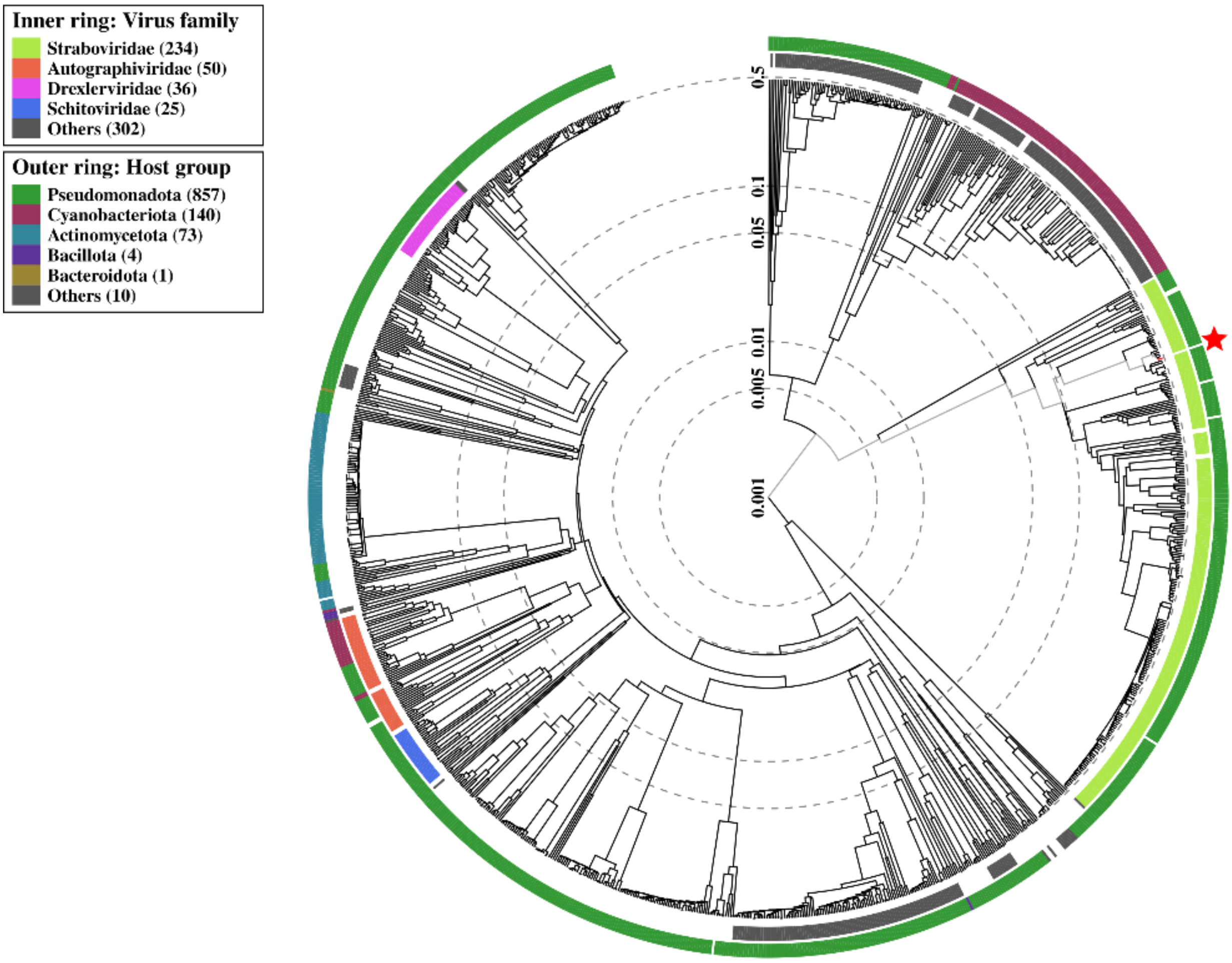

Figure S2: Proteomic tree generated by ViPTree of Enterobacter phage vB_EclM-HK6. Circular proteomic tree of Enterobacter phage vB_EclM-HK6, top BLASTn hits, and related phages of RefSeq genomes. (*) indicates Enterobacter phage vB_EclM-HK6.
